# CytoSpectre: a tool for spectral analysis of oriented structures on cellular and subcellular levels

**DOI:** 10.1186/s12859-015-0782-y

**Published:** 2015-10-26

**Authors:** Kimmo Kartasalo, Risto-Pekka Pölönen, Marisa Ojala, Jyrki Rasku, Jukka Lekkala, Katriina Aalto-Setälä, Pasi Kallio

**Affiliations:** Department of Automation Science and Engineering, Tampere University of Technology, BioMediTech, Tampere, Finland; University of Tampere, BioMediTech, Tampere, Finland; School of Medicine, University of Tampere, Tampere, Finland; School of Information Sciences, University of Tampere, Tampere, Finland; Heart Hospital, Tampere University Hospital, Tampere, Finland

**Keywords:** Orientation, Isotropy, Spectral analysis, Fourier transform, Power spectrum, Image analysis, Microscopy, Artificial images, Cardiomyocytes, Stretching

## Abstract

**Background:**

Orientation and the degree of isotropy are important in many biological systems such as the sarcomeres of cardiomyocytes and other fibrillar structures of the cytoskeleton. Image based analysis of such structures is often limited to qualitative evaluation by human experts, hampering the throughput, repeatability and reliability of the analyses. Software tools are not readily available for this purpose and the existing methods typically rely at least partly on manual operation.

**Results:**

We developed CytoSpectre, an automated tool based on spectral analysis, allowing the quantification of orientation and also size distributions of structures in microscopy images. CytoSpectre utilizes the Fourier transform to estimate the power spectrum of an image and based on the spectrum, computes parameter values describing, among others, the mean orientation, isotropy and size of target structures. The analysis can be further tuned to focus on targets of particular size at cellular or subcellular scales. The software can be operated via a graphical user interface without any programming expertise. We analyzed the performance of CytoSpectre by extensive simulations using artificial images, by benchmarking against FibrilTool and by comparisons with manual measurements performed for real images by a panel of human experts. The software was found to be tolerant against noise and blurring and superior to FibrilTool when analyzing realistic targets with degraded image quality. The analysis of real images indicated general good agreement between computational and manual results while also revealing notable expert-to-expert variation. Moreover, the experiment showed that CytoSpectre can handle images obtained of different cell types using different microscopy techniques. Finally, we studied the effect of mechanical stretching on cardiomyocytes to demonstrate the software in an actual experiment and observed changes in cellular orientation in response to stretching.

**Conclusions:**

CytoSpectre, a versatile, easy-to-use software tool for spectral analysis of microscopy images was developed. The tool is compatible with most 2D images and can be used to analyze targets at different scales. We expect the tool to be useful in diverse applications dealing with structures whose orientation and size distributions are of interest. While designed for the biological field, the software could also be useful in non-biological applications.

**Electronic supplementary material:**

The online version of this article (doi:10.1186/s12859-015-0782-y) contains supplementary material, which is available to authorized users.

## Background

Oriented features, such as various fibrillar structures of the cytoskeleton and the extracellular matrix, are frequent in biological systems but their analysis is often limited to subjective assessment by human experts [1]. Such procedures are not only typically too arduous to allow high-throughput studies but their reliability and repeatability are also questionable. Both optical techniques and software tools have been developed to obtain quantitative information in repeatable and automated manner. The software approach is advantageous in the sense that instead of using special instruments, the analyses can be performed for images captured with routinely used microscopy techniques. The proposed computational methods typically rely either on intensity-based segmentation, partial derivative approaches or spectral analysis. Separating individual objects from the image by segmentation is often difficult due to intensity variations and the presence of interfering high intensity features. Methods utilizing different forms of partial derivatives, on the other hand, detect the sharp changes in pixel intensity near the edges of oriented structures. The directions of intensity gradients can then be used as a measure of orientation in a particular image region. Methods of this family have been used to analyze e.g., myofiber arrangement in cardiac tissue [2], orientation of endothelial cells subjected to fluid flow [3], the organization of collagen fibers in the adventitia of arteries [4] and the orientation of fibrillar structures in plant cells [1]. Methods of the third class are markedly different, as instead of processing the images in the original spatial domain, they transform each image into an alternative frequency domain representation by decomposing the image into a spectrum of periodic components. This spectrum is called the power spectrum or power spectral density (PSD) of the image. By analyzing the PSD, one can estimate the orientation distribution of the different structures present in the image. Moreover, the PSD also contains information which can be related to the size of the structures. Spectral methods have been applied to the analysis of e.g., collagen and other fibers in different tissues and biomaterials [5–10], myocytes subjected to magnetic fields [11], the contractile cytoskeleton of stem cell derived cardiomyocytes [12, 13], neurite development [14] and keratoconic corneas [15]. In addition to these main classes of methods, approaches combining spatial and frequency domain analysis steps have been reported [16, 17].

Despite their numerous possible applications, few of the published methods are currently available in the form of user-friendly software. Previously published general purpose tools include the ImageJ [18] plug-ins FibrilTool [1] and OrientationJ [4], which allow the quantification of orientation distributions based on intensity gradients. Although highly useful in many applications, these methods still require significant manual operation in the form of selecting cells or regions of interest, or tuning of multiple parameters that lack any physical interpretations. Moreover, to the best of our knowledge, there are no publicly available tools utilizing spectral analysis for this purpose. In the case of images with heterogeneous content, such as complete cells with different subcellular structures visible, the possibility of separately examining features that fall into different spatial frequency bands could be highly beneficial. Such an approach would allow ‘unmixing’ of different image features that would otherwise all contribute to the final result of the analysis. This cannot be accomplished with spatial methods without significant image preprocessing steps. Such a tool could also take advantage of the capability to simultaneously analyze orientation and spatial frequency distributions, a feature of spectral analysis that has been mostly overlooked in previous studies. Spatial frequencies can be further transformed into wavelengths, which can be more easily interpreted to derive measures of object dimensions. Spectral methods are also relatively insensitive to noise [1] and can be flexibly adapted to different applications in a computationally efficient manner [9].

To enable spectral analysis of oriented features on both cellular and subcellular level with minimal user intervention, we developed a flexible software tool, CytoSpectre. CytoSpectre can be operated in highly automated fashion via a graphical user interface (GUI) on basic desktop computers without prior image processing expertise. In addition to the conventional approach of analyzing the whole power spectrum, dominated by the gross properties of the image, CytoSpectre extracts spectral regions representative of more detailed features such as fibrils or other intracellular structures. The obtained orientation and wavelength distributions and corresponding summary statistics can be easily exported in various formats for further study. To quantitatively measure the performance of CytoSpectre, we developed a simulation test bench capable of generating hundreds of artificial microscopy images with varying characteristics. We analyzed the performance of the software by an extensive set of simulations based on a large library of these computer-generated images and also by benchmarking against FibrilTool [1], a state-of-the-art orientation analysis tool. In the case of real images, performance of the software was validated by comparing computational and manual results for fluorescence and phase contrast images of human induced pluripotent stem cell (hiPSC) derived cardiomyocytes (hiPSC-CM) and peripheral sensory neurons (hiPSC-PSN). Finally, we demonstrated the functionality of the software in an actual experiment by studying the response of hiPSC-CMs to cyclic uniaxial stretching.

## Implementation

**Fig. 1:**
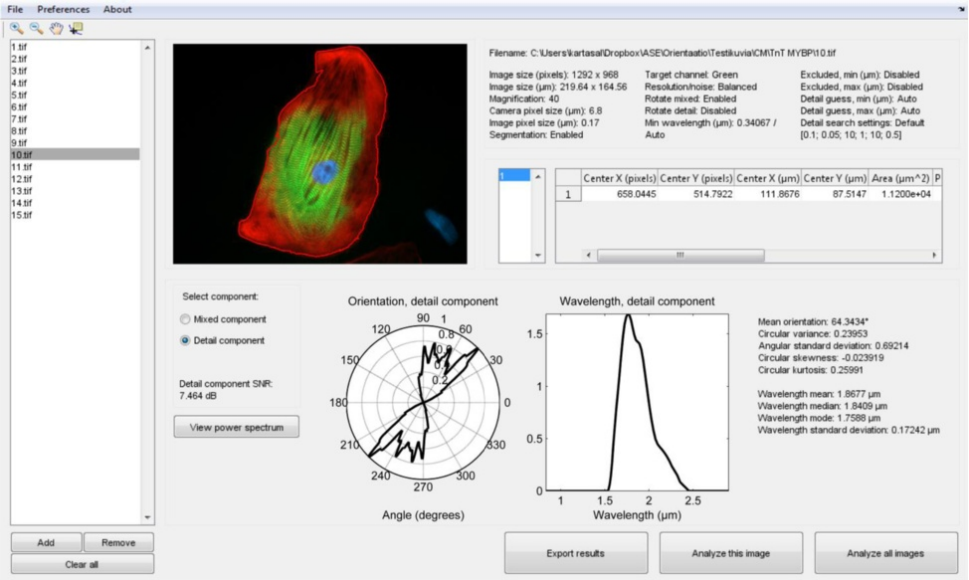
CytoSpectre graphical user interface. The GUI allows users to import (*far left*) and examine images (*upper left*, *cell outline obtained from a segmentation mask is shown in red*). Analysis settings and morphological parameters for each cell are shown in tables (*upper right*). The orientation and wavelength distributions are presented as plots and as summary statistics (*middle*). The GUI also features controls for running analyses and exporting results (*bottom right*)

**Fig. 2:**
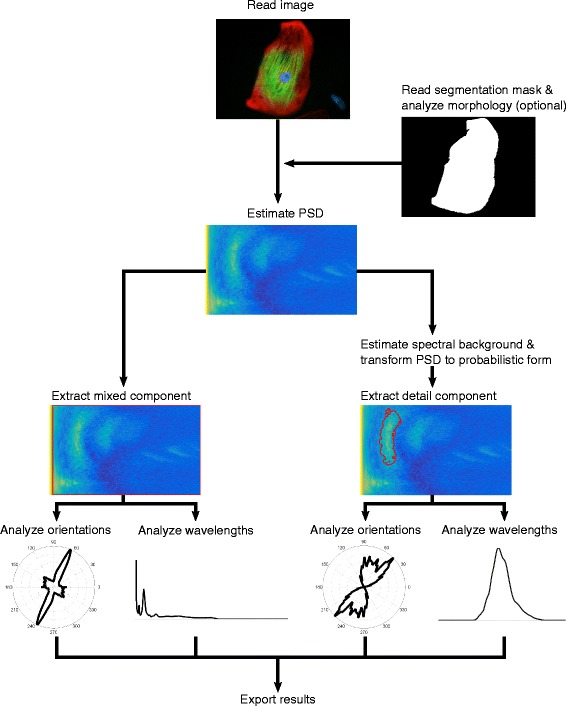
Flowchart of the analysis pipeline. At the beginning of an analysis run, images and (optional) segmentation masks are read into CytoSpectre. If segmentation masks are provided, descriptors of cellular morphology are computed before estimation of the PSD. Spectral regions of interest (*outlined in red*) corresponding to mixed and detail components are then extracted from the PSD. Orientation and wavelength analysis is performed for each component and the obtained results are typically exported for further study

CytoSpectre was developed using MATLAB R2014a (The MathWorks, Inc., Natick, MA, USA) and it can be used either under MATLAB or as a standalone 64-bit Windows 7 application. The standalone application may be used free of charge for the purpose of academic research even without a MATLAB license by installing the MATLAB Compiler Runtime library, which can be obtained automatically during installation. The installer for the standalone deployed application is provided in Additional file 1 and the MATLAB source codes in Additional file 2. Instructions for operating the software are given in the user’s guide in Additional file 3. CytoSpectre can be operated either via the MATLAB command line or via a graphical user interface. A screenshot of the CytoSpectre GUI during a typical analysis session is shown in Fig. 1. The GUI features controls for importing and examining images, customizing analysis settings, running the analysis and for exporting results. Analysis results are also presented in graphical form as plots. The computation time required for a single image of typical size (see the Results and Discussion section for details) on a basic laptop PC (equipped with an Intel Core™ i5 processor) was approximately 2–3 s in our experiments, allowing even large datasets to be analyzed without special hardware. The main analysis steps performed for each image are illustrated in Fig. 2 and described in the following sections. More detailed information concerning each step of the analysis can be found in the user’s guide and the supplementary information provided in Additional file 3 and Additional file 4, respectively.

### Importing images and selecting analysis settings

CytoSpectre is compatible with grayscale and RGB images in .tif, .jpeg, .gif., .bmp and .png image formats. In principle, 2D images obtained using any form of microscopy are supported. Only a single image is loaded into memory at a time, allowing users to examine large sets of images without particular system requirements in terms of available memory. CytoSpectre neither performs nor requires any particular preprocessing steps for the images. Before starting an analysis, the user needs to input the correct values for magnification, pixel size of the camera and in the case of RGB images, the color channel of interest. Magnification can also be detected automatically from the file names of the images if certain naming rules are followed. The physical size of the image pixels is then obtained by dividing the camera pixel size by magnification. A number of other settings controlling the analysis may also be adjusted but based on a sensitivity analysis using simulated images the results produced by the software are not overly sensitive to the exact values of these settings (see Additional file 4 and Additional file 5). More information on these settings is available in Additional file 3 and Additional file 4. Once suitable settings have been found, CytoSpectre allows them to be stored in a user profile file, which can then be loaded during later analysis sessions. This feature also makes it easy to store favorite settings suitable for different types of images.

### Importing cell segmentation data and analyzing cellular morphology

In addition to analyzing complete images as a whole, CytoSpectre allows the spectral analysis to be performed on a cell-by-cell basis. This can be accomplished by providing CytoSpectre with cell segmentation data in the form of mask images, along with the actual images. The mask images can be either binary images or label images. Binary images show background pixels in black and pixels belonging to cells in white. In that case, CytoSpectre assumes that each connected region of white pixels represents a single cell. Alternatively, the mask image can have labels for each cell, allowing the separation of touching cells. That is, in label images, background pixels have an intensity value of zero, pixels belonging to the ‘first’ cell have a value of one, pixels belonging to the ‘second’ cell have a value of two and so on. In addition to the spectral analysis, basic descriptors of cellular morphology, such as cell area, perimeter and eccentricity are computed for each cell. A large number of image processing and analysis tools, for example ImageJ [18] and CellProfiler [19], are available for performing the actual segmentation step. In some cases, the segmentation masks may even be formed manually. Since specialized tools fine-tuned for a particular application are often required for successful segmentation, we did not choose to include any single tool as an integral part of CytoSpectre. Instead, users can freely perform the segmentation step using tools they have found suitable and import the final results into CytoSpectre as mask images. However, the segmentation step is optional and in most cases it is probably sufficient to analyze the complete image as such, especially if only a single cell is present in the image.

### Spectral estimation

The PSD of the image is estimated using Welch’s method, also known as Weighted Overlapped Segment Averaging (WOSA) [20], with 50 % overlap between neighboring segments. The size of the segments, controlling the tradeoff between spectral resolution and stability, can be selected by the user from five different options: maximum resolution, high resolution, balanced, low noise or minimum noise. In most cases, the balanced setting is appropriate. Circularly symmetric Hann windowing is applied to each segment before computing its Discrete Fourier Transform (DFT) in order to reduce spectral leakage. If cell segmentation is enabled, background pixels are set to zero prior to spectral estimation. For more convenient processing during later analysis steps, the PSD is then transformed into polar coordinates.

### Separation of spectral components

CytoSpectre separates two components from the polar PSD, which we refer to as the mixed component and the detail component. The mixed component represents all features of the image present in the range of spatial frequencies dictated by upper and lower cutoff frequencies. By default, these frequency cutoffs are employed merely to exclude extremely low spatial frequencies, which typically only contain uninteresting, poorly resolved features [5, 6] or windowing artefacts [21]. However, the cutoff values may also be adjusted by specifying corresponding wavelength cutoffs. This may be desirable e.g., to exclude high-frequency noise or to focus the analysis on a particular wavelength range of interest. Analyzing the mixed component is appropriate for images having relatively homogeneous content such as a confluent layer of cells in a phase contrast micrograph. Inspecting the mixed component can also be a good starting point for any analysis.

**Fig. 3:**
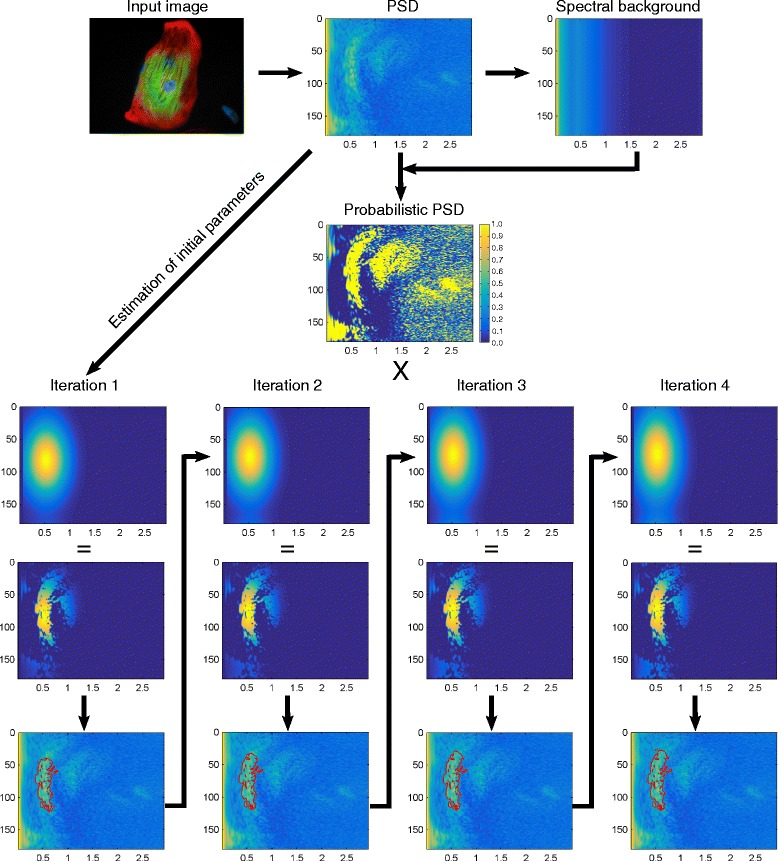
Flowchart of the detail component extraction procedure. An estimate of spectral background (*topmost right*) is first obtained by applying a smoothing operation on the PSD (*topmost middle*) of the input image (*topmost left*). The PSD is then converted to probabilistic form (*center*) based on the values of the PSD relative to spectral background. An initial 2D Gaussian ‘selector function’ (*iteration 1*, *top row*) is formed based on parameters estimated from the PSD. The 2D Gaussian is multiplied with the probabilistic PSD to obtain ‘posterior probabilities’ of belonging to the detail component region (*iteration 1*, *middle row*). A threshold of 0.5 is then applied to the ‘posterior’ to obtain the initial detail component region (*iteration 1*, *bottom row*; *region outlined in red*). The process then proceeds in iterative manner. Parameters for the ‘selector function’ used during the second iteration (*iteration 2*, *top row*) are estimated based on the detail component spectral region obtained during the first iteration. The same steps are then repeated during each iteration until the parameters of the ‘selector function’ reach convergence at a certain threshold (in this case after four iterations) or the maximum number of allowed iterations is reached. The final estimate of the detail component spectral region is retained for further analysis steps. The x-axis in all of the spectral plots indicates spatial frequency in μm^−1^ while the y-axis indicates orientation in degrees. The values of the PSD, spectral background and the detail component region plots are given in arbitrary units. The values of the probabilistic PSD, the Gaussian ‘selector function’ and the ‘posterior’ plots are given as probabilities, as indicated by the color bar on the right-hand side of the probabilistic PSD

The detail component, on the other hand, represents some particular group of structures with a limited range of spatial frequencies and/or orientations, such as intracellular fibrils of given size. If present in the image, such structures are reflected in the power spectrum as more or less contiguous regions of high intensity relative to background. The aim of the detail component extraction procedure is to separate these regions from the rest of the power spectrum, allowing the user to obtain results that more specifically represent the structures of interest. The main advantage of this approach is that it allows the exclusion of interfering spectral components arising from, for example, the contours of a cell’s cytoplasm or from variations in fluorescent background signal across the image. CytoSpectre uses an iterative method, bearing some resemblance to a Bayesian classifier and segmentation algorithms based on region growing, to separate the spectral region corresponding to the detail component. If an estimate of the size of the targets of interest is available a priori, this information can be provided as a wavelength guess to guide the algorithm. The procedure, illustrated in Fig. 3, consists of first forming an estimate of spectral background using an approach based on smoothing the actual PSD estimate. The background estimate is utilized to obtain a probabilistic representation of the PSD. ‘Posterior probability’ of belonging to the detail component spectral region is obtained for each spectral element by multiplying the probabilistic PSD with a 2D Gaussian ‘selector function’, whose role is similar to a prior in a Bayesian classifier. Initial parameters of the 2D Gaussian are estimated based on the circular statistics of the PSD and a 1D peak fitting step. An initial estimate of the detail component spectral region is then formed by thresholding the ‘posterior probabilities’. The region is then adapted to the actual shape of the spectral area of interest by modifying the 2D Gaussian at each iteration, based on the circular statistics of the spectral region extracted during the previous iteration. Technical details of the procedure are described in Additional file 4.

### Orientation and wavelength analysis

After the mixed and detail spectral component have been extracted from the polar power spectrum, their spectral elements are averaged over all spatial frequencies and all orientations to obtain one-dimensional distributions of orientations and spatial frequencies, respectively. For the orientation distribution, summary statistics are computed using the functions provided by the CircStat toolbox [22]. In addition to the mean orientation, given in degrees, they include circular variance and angular standard deviation, which are two alternative measures of isotropy, as well as circular skewness and circular kurtosis, which are indicative of the symmetry and peakedness of the orientation distribution, respectively. More information on these parameters is available in Additional file 3 and Additional file 4. The spatial frequency distribution is first transformed into a wavelength distribution, which in turn reflects the size distribution of target structures, and descriptive statistics (mean, median, mode and standard deviation) are then computed. The wavelengths are given in micrometers by default, but the preferred unit can be selected by the user.

### Examining and exporting results

After finishing an analysis run, the estimated orientation and wavelength distributions can be examined as plots via the CytoSpectre GUI together with summary statistics. If segmentation is enabled, allowing analysis of cellular morphology, the corresponding statistics are also shown in tabular form for each cell. The power spectrum can also be examined visually in order to spot interesting spectral features, which may then be extracted by tuning the wavelength cutoffs and/or the expected detail component wavelength range. CytoSpectre allows the user to export the analysis results in a variety of formats. Summary statistics can be easily exported to spreadsheets or plain text files for further study. The analysis settings used to obtain the results are also stored in these files. In addition, the user can export plots of the estimated orientation and wavelength distributions as images for visualization purposes. It is also possible to export the actual orientation and wavelength distribution values in plain text files, allowing customized analysis pipelines to be developed utilizing external software.

## Results and discussion

### Performance on synthetic phase contrast microscopy images of cell clusters

The performance of orientation analysis algorithms has typically been assessed using simple computer-generated test images featuring randomly placed Gaussians, ellipsoids or lines [1–3, 5, 7, 9, 10, 15]. In line with this traditional approach, a MATLAB script was used to generate 1000 synthetic images featuring randomly placed two-dimensional Gaussian functions as targets, resembling clusters of cells in a phase contrast microscopy image. The sizes, aspect ratios and orientations of the Gaussians were obtained by sampling from normal and von Mises distributions with parameters varying from image to image to create a diverse set of test images. The approximate mean width of these artificial cells varied between 10 and 50 μm from image to image. In the remainder of the article, we refer to this set of images as the ‘cell cluster dataset’. The test images were then analyzed using CytoSpectre with and without different types of image quality degradations and the estimated values were compared with the true values. In the comparisons, we used the values estimated for the mixed component, as the sizes of the targets in a single image were not particularly limited to a certain band of wavelengths and the images do not contain any other structures besides the targets of interest. To simulate real-life processes leading to image quality degradation in microscopy, signal-dependent Poisson distributed noise and additive white Gaussian noise were added into the synthetic images [23]. In addition to noising, blurring was applied by filtering the images with a Gaussian filter to simulate the effects of imperfect focus, leading to loss of sharp details in the images [1]. Each of the degradation operations was performed for all 1000 images and the images were then analyzed by CytoSpectre to quantify the effects of degraded image quality. The analysis settings used for the original images were not adjusted in any way for the degraded images. A detailed description of the synthetic image generation and degradation process is given in Additional file 4 while full numerical data and the used analysis settings from these experiments are provided in Additional file 6.

First, we analyzed the images without any degradation. The results of this experiment are summarized in Table 1. For each parameter, we calculated the means and standard deviations of the errors. To allow comparisons between different parameters having different numerical values, we also computed the corresponding statistics for normalized errors, which were obtained by normalizing the errors relative to the full range of the true values of each parameter. Moreover, we computed Pearson’s linear correlation coefficient between the true and estimated values for each parameter. In the case of the mean orientation, which is the only parameter defined on a circular scale, only the absolute errors were analyzed. The results show that the mean orientation could be estimated with an accuracy that is highly likely to be sufficient for most applications since errors on the order of a few degrees are probably overshadowed by biological or technical variation in most experiments. It should be noted that we did not exclude even highly isotropic test images from this analysis. In the most extreme cases, the structures are so isotropic that even the true mean orientation is not really indicative of any preferred orientation which can result in large, more or less random errors for such images. The obtained orientation error values can therefore be seen as rather pessimistic. The other orientation and wavelength parameters, except for circular skewness, exhibit relatively high mean errors, indicating that the numerical values of these parameters should be interpreted with caution. However, the linear correlation coefficients are acceptable for all parameters, ranging from ~0.42 to ~0.95, which indicates that the errors are mostly attributable to systematic bias. This means that the estimated values can still be useful in experiments where a point of comparison is available. For example, values obtained from samples treated with a chemical of interest could be compared to a non-treated control sample. As such a comparative experimental setup including control samples is typical for many biological assays we feel that the high systematic bias in most of the estimated parameters is not a major issue, as long as the user acknowledges the fact that the numerical values should not be interpreted without a point of reference. Moreover, even in the absence of any systematic bias, interpreting many of the parameters (e.g., circular variance) directly based only on their numerical values would probably be possible only for a statistics expert with previous experience of working with such measures. In other words, as the answer to the question of whether a particular value of circular variance, for example, is ‘high’ or ‘low’ is usually relative and depends on the case at hand, strong linear dependence between true and estimated values is much more important than the amount of systematic bias.

**Table 1:** Performance on simulated images of cell clusters

	Error mean ± std	Normalized error mean ± std	Pearson’s r
Mean orientation	1.5534° ± 3.3967°	-	-
Circular variance	0.2384 ± 0.2162	0.2384 ± 0.2162	0.4156
Angular std	0.4042 ± 0.2632	0.2858 ± 0.1861	0.4230
Circular skewness	−0.0001 ± 0.0312	−0.0002 ± 0.0578	0.4686
Circular kurtosis	−0.3930 ± 0.2209	−0.3272 ± 0.1840	0.4980
Wavelength, mean	21.9955 μm ± 5.5363 μm	0.5281 ± 0.1329	0.9532
Wavelength, median	24.4053 μm ± 6.1943 μm	0.5703 ± 0.1447	0.9262
Wavelength, mode	31.7249 μm ± 10.1070 μm	0.7023 ± 0.2237	0.4770
Wavelength, std	2.1006 μm ± 3.6847 μm	0.1104 ± 0.1936	0.4722

Errors (mean ± standard deviation) and Pearson’s linear correlation coefficient between true and estimated values for non-degraded simulated images of the cell cluster dataset (*N* = 1000) are shown for each parameter. Normalized errors were obtained by dividing the errors by the full range of true values. The normalized error and linear correlation coefficient are not applicable to the mean orientation. Mean orientation error is given as absolute error in degrees

**Fig. 4:**
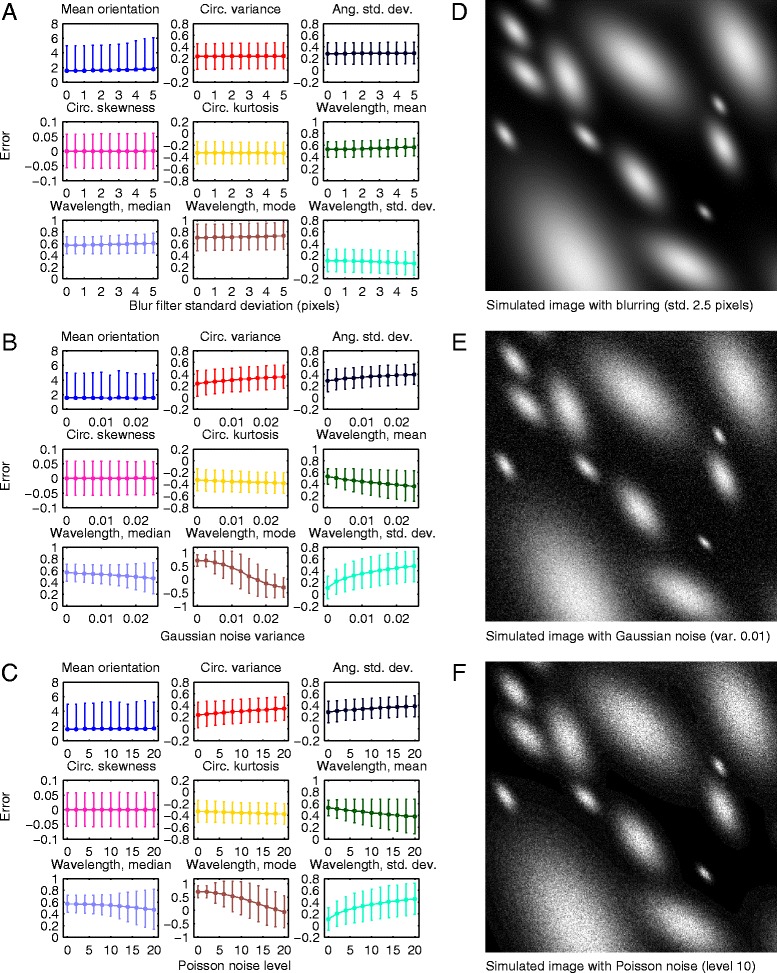
Simulation results for the cell cluster dataset. Errors (mean ± standard deviation) between estimated and actual values for each parameter using images of the cell cluster dataset (*N* = 1000). The errors are shown for images blurred with Gaussian kernels having standard deviations from 0 to 5 pixels (**a**), images with additive white Gaussian noise having normalized variances from 0 to 2.5 % (**b**) and images with Poisson distributed noise at levels 0–20 (**c**). For the mean orientation, the errors are absolute and given in degrees. For the other parameters, the errors were normalized by the range of true values. Examples of images with moderate blurring (std 2.5 pixels) (**d**), Gaussian noise (var. 1 %) (**e**) and Poisson noise (level 10) (**f**) are shown

Images with different levels of blurring were obtained by filtering with Gaussian kernels having different standard deviation values, ranging from 0.5 to 5 pixels. Errors in the estimated parameter values obtained at different levels of blurring are plotted in Fig. 4a. An example of a moderately blurred image is shown in Fig. 4d. The effect of blurring on the results was negligible for all of the parameters with both the mean and the standard deviation of the errors staying almost constant. Images with different levels of additive white Gaussian noise were obtained by adding noise with zero mean and normalized variance ranging from 0.25 to 2.5 %. These values correspond to approximate standard deviations of 13 and 40, respectively, on the absolute 8-bit intensity scale (0–255). Errors in the estimated parameter values obtained in the presence of different amounts of Gaussian noise are plotted in Fig. 4b while an example of an image with moderate Gaussian noise is shown in Fig. 4e. The estimates of parameters describing the orientation distribution, especially the mean orientation and circular skewness, are able to tolerate even extreme amounts of Gaussian noise with only a moderate increase in error. The wavelength statistics, on the other hand, are more sensitive towards Gaussian noise. In the case of the mean, median and mode of the wavelength distribution, the mean errors actually decrease with increased noise but at the same time, the standard deviation of the error increases, indicating increased random variation. Finally, images with different levels of signal-dependent Poisson noise were obtained as in [23] by using scaling factors ranging from 2 to 20 during the noise generation process. Errors in the estimated parameter values obtained in the presence of different levels of Poisson noise are plotted in Fig. 4c while an example of an image with moderate Poisson noise is shown in Fig. 4f. Overall, the effect of Poisson noise on the results is very similar to that of Gaussian noise. It should be noted, that the more extreme amounts of noise and blurring tested in these experiments represent a worst case scenario, which would rarely be encountered in practice unless there was a technical problem with the imaging system.

### Performance on synthetic fluorescence microscopy images of cells with intracellular fibrillar structures

In addition to the more traditional performance evaluation with simple 2D Gaussian targets, the performance of the software was also evaluated with more sophisticated test images containing 2D Gaussian subunits organized into intracellular fibrillar structures. SimuCell [24] was used to generate synthetic images of cells and nuclei, and an in-house developed MATLAB algorithm was utilized to construct the fibrillar structures within the cells. As a result, a library of 1000 images with characteristics imitating those of fluorescence microscopy images captured from cells with immunostained intracellular fibrils was generated. Similarly to the cell cluster dataset, random sampling from normal and von Mises distributions was used to generate images with different fibril orientations and isotropy, different numbers of fibrils and fibril subunits, and different fibril subunit sizes and spacing. In the remainder of the article, we refer to this set of images as the ‘fibril dataset’. In the case of this dataset, the targets of interest are present within a particular range of wavelengths and there are interfering features in the image, namely the nucleus and the cell borders. This allowed us to try focusing the analysis to the targets by using the detail component values rather than the mixed component, which would also include contributions from the interfering structures. The image quality degradation experiments performed for the cell cluster dataset were repeated for the fibril dataset. Whenever the detail component could not be detected, the image in question was excluded from the analysis. The total number of excluded images varied between zero and four (corresponding to 0–0.4 % of all images) depending on the type and severity of the degradations. The largest number of four exclusions was actually observed for the non-degraded images, which is probably a consequence of the less natural spectra of these images. A detailed description of the synthetic image generation and degradation process is given in Additional file 4 while full numerical data and the used analysis settings from these experiments are provided in Additional file 6.

Similarly to the cell cluster dataset, the images of the fibril dataset were first analyzed without any image quality degradation. The results of this experiment are summarized in Table 2. The errors of the estimated mean orientations were slightly higher than for the cell cluster dataset but the mean error is still less than two degrees. This is again a pessimistic value, as even the most isotropic cells are included in the analysis. The other orientation distribution parameters exhibited low errors as well, with all of the normalized mean errors below 5 %. Linear correlation coefficients between the true and estimated circular variance, angular standard deviation and circular kurtosis were rather high, ranging from ~0.53 to ~0.65, while circular skewness exhibited a moderate correlation of ~0.34. The decrease of systematic bias relative to that observed for the cell cluster dataset and the mixed component is probably explained by the ability of the detail component to capture only the relevant part of the power spectrum, which appears to be representative of the targets of interest, while decreasing the contributions of irrelevant parts of the spectrum. In the case of the wavelength parameters, the strong improvement was limited to the wavelength mode. The wavelength mode had a mean error of only 6.1 nm (with the size of the targets being on the order of a few micrometers) or 0.54 % of the full range of true values. As the simulated images have a pixel size of 170 nm, this level of error indicates subpixel accuracy for the majority of the images. A high linear correlation coefficient of ~0.78 also indicated that the estimated wavelength mode reflects the true values reliably. The mean, median and standard deviation parameters of the wavelength distribution did not show similarly improved results, however, with weak or non-existent correlations ranging from ~0.09 to ~0.19. Based on these results, when using the detail component to separate targets of interest from the image, the orientation parameters may be used with confidence but the wavelength statistics, excluding the mode, should be treated with caution. The mode of the wavelength distribution, however, reliably indicates the typical size of the targets of interest. This was expected, as the mode has been used previously e.g., as a measure of the sarcomere length of cardiomyocytes [12].

**Table 2:** Performance on simulated images of cells with intracellular fibrillar structures

	Error mean ± std	Normalized error mean ± std	Pearson’s r
Mean orientation	1.8128° ± 7.1196°	-	-
Circular variance	−0.0119 ± 0.1427	−0.0119 ± 0.1427	0.5305
Angular std	0.0149 ± 0.1982	0.0106 ± 0.1401	0.5414
Circular skewness	0.0008 ± 0.0311	0.0015 ± 0.0616	0.3432
Circular kurtosis	−0.0509 ± 0.1808	−0.0453 ± 0.1609	0.6500
Wavelength, mean	0.3886 μm ± 0.5756 μm	0.3809 ± 0.5642	0.1681
Wavelength, median	0.2109 μm ± 0.5283 μm	0.2020 ± 0.5061	0.1978
Wavelength, mode	0.0061 μm ± 0.2002 μm	0.0054 ± 0.1778	0.7817
Wavelength, std	0.4912 μm ± 0.4384 μm	2.2042 ± 1.9671	0.0936

Errors (mean ± standard deviation) and Pearson’s linear correlation coefficient between true and estimated values for non-degraded simulated images of the fibril dataset (*N* = 996) are shown for each parameter. Normalized errors were obtained by dividing the errors by the full range of true values. The normalized error and linear correlation coefficient are not applicable to the mean orientation. Mean orientation error is given as absolute error in degrees

**Fig. 5:**
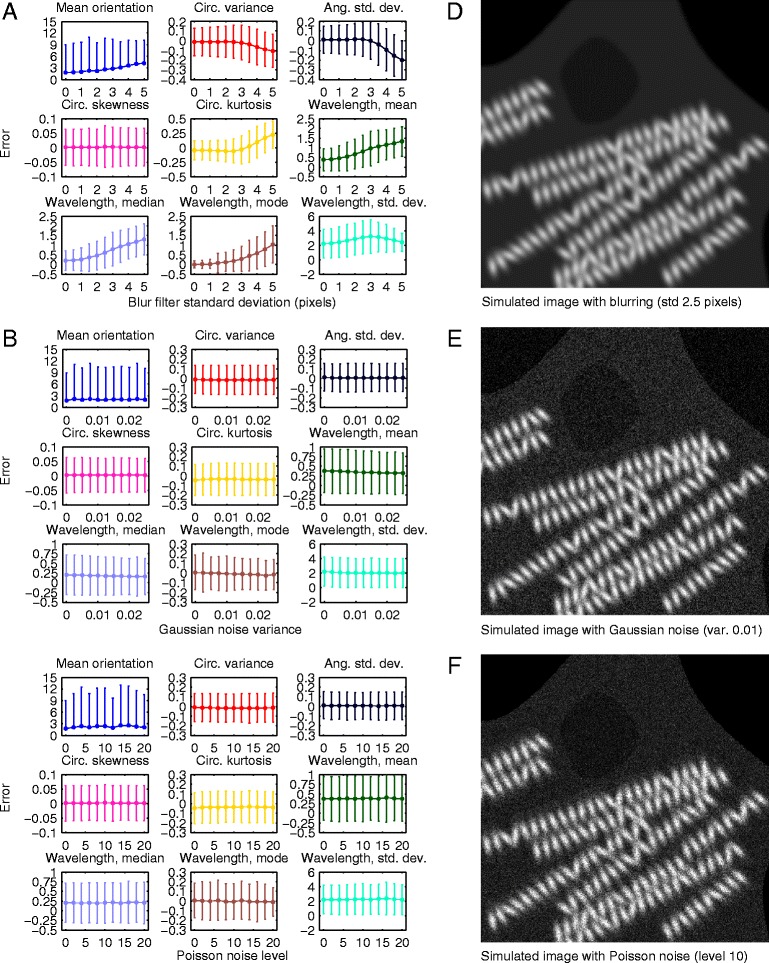
Simulation results for the fibril dataset. Errors (mean ± std) between estimated and actual values for each parameter using images of the fibril dataset (*N* = 1000). The errors are shown for images blurred with Gaussian kernels having standard deviations from 0 to 5 pixels (**a**), images with additive white Gaussian noise having normalized variances from 0 to 2.5 % (**b**) and images with Poisson distributed noise at levels 0–20 (**c**). For the mean orientation, the errors are absolute and given in degrees. For the other parameters, the errors were normalized by the range of true values. Examples of images with moderate blurring (std 2.5 pixels) (**d**), Gaussian noise (var. 1 %) (**e**) and Poisson noise (level 10) (**f**) are shown

The effects of blurring, Gaussian noise and Poisson noise on the results of the fibril dataset are visualized in Fig. 5a-c with example images shown in Fig. 5d-f. As opposed to the cell cluster dataset, serious blurring increased the errors of most parameters. This is natural, as the fibril subunits are much smaller and closer to each other than the Gaussian targets of the cell cluster dataset. Sharper images are therefore required to properly capture these detailed structures. However, the errors only start to increase markedly after the blur kernel standard deviation is increased beyond 2–3 pixels while less severe blurring is still tolerated well. Moreover, increased blurring smoothly increases the errors rather than causing sudden, complete breakdown of the detail component detection procedure. Tolerance against even extreme amounts of Gaussian and Poisson noise, on the other hand, is very high as none of the estimated parameters exhibit any clear sensitivity to either type of noise. The adverse effects of severe noise observed in the case of the cell cluster dataset are probably avoided due to the ability of the detail component to again extract only the relevant part of the power spectrum, decreasing the relative contribution of noise.

### Validation using real fluorescence microscopy images of hiPSC-derived cardiomyocytes

**Fig. 6:**
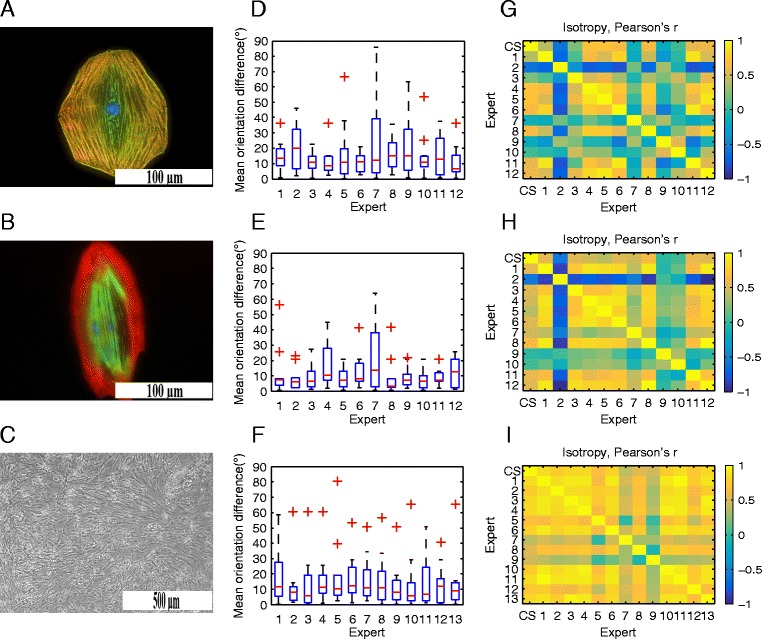
Results for real images. Examples of hiPSC-CMs from validation set 1 (**a**) and validation set 2 (**b**), imaged using fluorescence microscopy, are shown. An example of hiPSC-PSNs, from validation set 3, imaged using phase contrast imaging is shown in (**c**). The distributions of absolute differences in degrees between mean orientations estimated by CytoSpectre and by each human expert are plotted in (**d**), (**e**) and (**f**) for images of validation set 1 (*N* = 11), validation set 2 (*N* = 10) and validation set 3 (*N* = 10), respectively. If ¼ or more of the human experts were unable to specify the mean orientation for an image, the image was excluded from this analysis. The red line is the median, the edges of the blue box are the 25th and 75th percentiles and the whiskers extend to 1.5 times the interquartile range (corresponding to approximately 99.3 % coverage for normally distributed data). Values beyond these limits are considered outliers and are plotted as red crosses. Pearson’s linear correlation coefficients between anisotropy rankings estimated by human experts and circular variance values estimated by CytoSpectre (CS) for each human-human or CS-human pair are shown as correlation matrices in (**g**), (**h**) and (**i**) for images of validation set 1 (*N* = 15), validation set 2 (*N* = 15) and validation set 3 (*N* = 15), respectively

Two sets of real images of hiPSC-CMs with different immunofluorescent stainings were used to validate the performance of CytoSpectre with fluorescence microscopy images. Spontaneously beating hiPSC-CMs were differentiated by END-2 co-culture method [25] from hiPSCs reprogrammed from skin fibroblasts [26]. Differentiated hiPSC-CMs appeared as beating clusters in co-culture after 15 days of initiation of differentiation. These clusters were cut, dissociated and cultured as single hiPSC-CMs [27]. Cultured cells were fixed and stained by immunocytochemistry with cardiac specific antibodies binding on the sarcomere structures of hiPSC-CMs. Samples were imaged with Zeiss AxioScope.A1 fluorescence microscope with Zeiss AxioCam MRc5 using 2 × 2 binning and an effective pixel size of 6.8 μm. The first set (validation set 1) included 15 images at 20× and 40× magnifications, stained with DAPI and antibody labels for cardiac Troponin T and α-actinin. An example image from this set is shown in Fig. 6a. The second set (validation set 2) included 15 images at 40× magnification, stained with DAPI and labels for cardiac Troponin T and Myosin binding protein C3. In these images, the striated sarcomere patterns were clearly visible. An example image is shown in Fig. 6b. A detailed description of the experimental methods is given in Additional file 4. Validation sets 1 and 2 were analyzed by 12 human experts, i.e., researchers working with hiPSC-CMs, based only on visual examination of the images. The experts were asked to give an estimate of the mean orientation of the intracellular myofibrils present in the images. The experts also had the option of leaving the mean orientation unspecified in the case of cells exhibiting a high degree of isotropy (i.e., lacking any preferred orientation). Some experts also specified multiple main orientations for a single cell, in which case circular averaging was used to obtain a single mean orientation value for each image. In addition to estimating the mean directions, the experts were asked to rank the images of each set based on the anisotropy of the myofibrils. We limited ourselves to analyzing the mean orientation and anisotropy only, since estimating the wavelength parameters (e.g., sarcomere length) would have been highly challenging based on visual examination and the value of such results would have been questionable. The images were then analyzed using CytoSpectre and the computational results were compared with the expert evaluation. Full numerical data and analysis settings for these experiments are provided in Additional file 7.

The myofibrils visible in the images of the validation set 1 were not limited to any clearly specific band of wavelengths in the power spectrum. We therefore analyzed the mixed component using a relatively broad wavelength range from 1 to 5 μm. This selection allowed us to exclude noise and irrelevant structures present at high spatial frequencies (i.e., short wavelengths) as well as structures much larger than the myofibrils, such as the nucleus and the shape of the entire cell. The mean orientations estimated by CytoSpectre were then compared with the manual estimates of each expert. We excluded the most isotropic images from this analysis, since mean orientation is not a meaningful quantity for highly isotropic cases. If ¼ or more of the human experts were unable to specify the mean orientation for an image, the image was excluded. Based on this criterion, 11 images were retained. The distributions of absolute differences between manual and computational mean orientations are visualized for each expert in Fig. 6d. The combined difference averaged over all experts and all images was 15.6° ± 14.6° (mean ± standard deviation). This can be seen as an acceptable result in view of psychological studies which indicate that humans may overestimate or underestimate angles by up to 10° [28, 29]. There was also considerable variation from expert to expert. For example, the difference between CytoSpectre and expert 3 was only 10.6° ± 5.9° while comparison with expert 7 produced a difference of 22.8° ± 24.8°. This variation clearly underlines the potential benefits of automated, objective analysis and casts doubts on the reliability of manual measurements relying on a single human observer, as is often the case.

We also analyzed the correlation between the circular variance values estimated by CytoSpectre and the ranking of images performed by the human experts, based on the perceived degree of anisotropy of the myofibrils. We computed Pearson’s linear correlation coefficient between the values estimated by CytoSpectre and by each human expert as well as between each possible pair of humans. All 15 images were used for this analysis. The results are visualized as a heatmap of correlation coefficients in Fig. 6g. The linear correlation coefficient between the values estimated by CytoSpectre and the rankings performed by human experts was 0.30 ± 0.43 (mean ± standard deviation) while the linear correlation coefficient between human experts was only 0.18 ± 0.50. While the mean correlation coefficient of 0.30 is rather low, there is considerable variation from expert to expert, similarly to the mean orientation estimates. Many of the human analysts, especially experts 1, 4, 5, 6, 8, 11 and 12 agree with the computational results much more strongly with correlation coefficients of approximately 0.40, 0.71, 0.70, 0.63, 0.65, 0.59 and 0.60, respectively. The remaining experts tend to disagree not only with CytoSpectre but also with each other, highlighting the diversity of ways of perceiving the images and performing scoring. In summary, these results seem to indicate that it is more likely for a randomly selected human expert to agree with the computational results than with another randomly selected human expert. Moreover, the software was able to estimate circular variance values which corresponded well with the majority of manual rankings, while the observed expert-to-expert variation again confirmed the need for objective analysis methods.

Next, we analyzed the second set of fluorescence microscopy images to evaluate the compatibility of the software with different immunofluorescent stainings. The staining used for the images of validation set 2 highlighted the characteristic striated patterns of the myofibrils (see Fig. 6b). The striations reflect the repeating structure of sarcomeres, which have a size of approximately 2 μm and are delimited by protein structures known as Z-disks [12]. In the power spectrum, sarcomeres appear as a prominent peak constrained within a narrow range of spatial frequencies close to 0.5 μm^−1^ (or equivalently within a narrow range of wavelengths close to 2 μm). Based on this information about the target structures, we analyzed the detail component using an expected wavelength range of 1.5 to 2.5 μm. Computational results were compared with manual results similarly to the validation set 1 images. Results of the mean orientation comparison are shown in Fig. 6e. Ten images with sufficient anisotropy were retained for this analysis based on the same criterion as with validation set 1. For these images, the difference in computationally and manually estimated mean orientations was 11.3° ± 12.0° (mean ± standard deviation) when averaged over all images and experts. The variation from expert to expert was again considerable, with the lowest mean difference of 8.1° ± 6.6° observed with expert 5 and the largest mean difference of 20.1° ± 20.0° observed again in the case of expert 7. Results of the correlation analysis for computationally and manually estimated measures of isotropy, performed for all 15 images, are shown in Fig. 6h. These results are very similar to the values observed for validation set 1, with Pearson’s linear correlation coefficient between circular variance values estimated by CytoSpectre and rankings performed by human experts having the value of 0.36 ± 0.36 (mean ± standard deviation). The corresponding value between human experts was 0.30 ± 0.54. Again, the relatively low mean linear correlation coefficients are largely explained by the remarkable variation among the human experts. The general pattern of the correlation heatmap is similar to the images of validation set 1 i.e., the same experts tended to agree and disagree with the computational results as with the first dataset. These results show that the detail component extraction procedure functions properly also in the case of real images and produces results that most human experts agree with relatively well. Moreover, these results support the idea that CytoSpectre is not limited to a particular fluorescent label but can be used in combination with different stainings.

### Validation using real phase contrast microscopy images of hiPSC-derived peripheral sensory neurons

A third set of real images (validation set 3), depicting hiPSC-PSNs, was used to validate the performance of CytoSpectre with a different microscopy technique, namely phase contrast imaging. The hiPSC-PSNs were differentiated from hiPSCs using the methods described in [30, 31]. Differentiated hiPSC-PSNs formed neurospheres which were cut, dissociated and plated on PA6 feeder cells. Samples were imaged with Nikon Eclipse TS100 phase contrast microscope with Imperx IGV-B1620M camera having a pixel size of 7.4 μm. This validation set included 15 images at 10× magnification. An example image is shown in Fig. 6c. A detailed description of the experimental methods is given in Additional file 4. Validation set 3 was analyzed by 13 human experts in a manner similar to validation sets 1 and 2. That is, the experts were asked to visually estimate the mean orientation of the clusters of neurons and to rank the images based on the perceived anisotropy of the cells. Full numerical data and analysis settings for these experiments are provided in Additional file 7.

Since the targets in these images are not clearly limited to a specific range of wavelengths and no significant interfering structures such as nuclei are visible, we analyzed the mixed component using the default settings for cutoff wavelengths. Ten images were retained for the comparison between computationally and manually estimated mean orientations based on the same exclusion criterion employed for the images of validation sets 1 and 2. The distributions of differences between mean orientations estimated by CytoSpectre and the human experts are shown in Fig. 6f. The difference averaged over all images and experts was 15.4° ± 17.4° (mean ± standard deviation), with the smallest mean difference of 11.9° ± 14.9° observed with expert 9 and the highest mean difference of 19.8° ± 23.9° observed with expert 5. Results of the correlation analysis for the estimated isotropy are shown in Fig. 6i. This analysis was again performed for all 15 images. The degree of agreement over the isotropy was high both between CytoSpectre and human experts and also between pairs of humans. Pearson’s linear correlation coefficient between computational and manual values was 0.72 ± 0.15 (mean ± standard deviation). The correlation coefficients between human experts were again slightly lower with a corresponding value of 0.67 ± 0.24. Overall, the results indicate that CytoSpectre is not limited to fluorescence microscopy but is also suitable for phase contrast imaging. Moreover, we have no theoretical or practical reasons to doubt the compatibility of the software with images captured using other 2D microscopy techniques, although only fluorescence and phase contrast images have been systematically evaluated thus far. This experiment also confirmed that the results obtained on a subcellular level for the images of cardiac myofibers can be generalized to images with different cell types and structures on a different scale.

### Comparison with existing software

As we are not aware of any existing freely available software aimed for spectral analysis of microscopy images, we benchmarked CytoSpectre against FibrilTool [1], a recently published orientation analysis plug-in for the popular ImageJ [18]. While FibrilTool cannot be used to estimate any wavelength statistics, we could still compare the orientation analysis capabilities of the two methods. FibrilTool allows the estimation of mean orientation and anisotropy of structures within user-specified regions of interest (ROI) on the basis of nematic tensors. For each image, we used an ImageJ macro to select the complete image as a ROI, because CytoSpectre analyzes the whole images as well. The default line width setting of one was used for all images, as suggested in the FibrilTool paper [1]. We applied FibrilTool in this manner for the first 100 images from each synthetic image dataset first without any degradation and then with moderate amounts of blurring (Gaussian kernel standard deviation 2.5 pixels), Gaussian noise (normalized variance 1 %) or Poisson noise (level 10). The total number of images to analyze was thus 400 per dataset, i.e., 800 in total. We used these subsets of images instead of the complete datasets because FibrilTool requires manual operation and analyzing 800 images in total, instead of 8000 images, was still feasible. The mean orientations estimated by FibrilTool were adjusted to follow the CytoSpectre angle convention (0°–180°), allowing a direct comparison. In the case of the fibril dataset, we observed that FibrilTool often detects the orientation parallel to the fibril subunits as the main orientation. This orientation is perpendicular to the actual orientation of the fibrils and we noted that the mean orientation error can be lowered by applying a correction of 90°. This correction was thus performed for all main orientations estimated by FibrilTool for this dataset. For the synthetic images of cell clusters, this adjustment was not necessary due to the absence of such problematic structures in these images. In contrast to CytoSpectre, FibrilTool does not estimate the circular variance for each ROI but instead an anisotropy index, which is a measure of anisotropy rather than isotropy. However, as both measures are defined in the range of 0 to 1, we simply obtained an isotropy index for each ROI as the complement of the corresponding anisotropy index (i.e., unity minus the anisotropy index). The resulting values are then directly proportional to circular variance, even though the numerical values of the two measures are not necessarily identical. Full FibrilTool outputs for the synthetic images are provided in Additional file 8.

Next, the results obtained by CytoSpectre in the manner described in the previous sections were compared with the results produced by FibrilTool. We again used the mixed component parameters in the case of the cell cluster dataset and the detail component parameters for the fibril dataset. For each dataset and type of image degradation, we calculated the mean and standard deviations of the absolute main orientation errors in degrees for both CytoSpectre and FibrilTool. Paired, two-sided Wilcoxon signed rank tests were performed for each case to evaluate the statistical significance of any differences between the orientation errors produced by the two tools. Similarly for each dataset and degradation type, we computed Pearson’s linear correlation coefficient between the true circular variance and the estimated measure of isotropy i.e., circular variance in the case of CytoSpectre and isotropy index in the case of FibrilTool. The resulting values are shown in Table 3 for the cell cluster dataset and in Table 4 for the fibril dataset. In the case of non-degraded or blurred images of the cell cluster dataset, FibrilTool produces main orientation estimates with slightly lower mean error than CytoSpectre. However, in both cases the difference is less than one degree, which probably does not have any practical significance in most applications. Moreover, in the case of the non-degraded images, the difference is not statistically significant at the 1 % significance level. As observed also in the image quality degradation experiment for both synthetic image datasets, the main orientation estimates of CytoSpectre are almost unaffected by blurring or noise. The corresponding estimates of FibrilTool are more sensitive towards image quality degradation, although in the case of the cell cluster dataset, blurring is tolerated well. In the case of added noise and for the fibril dataset in general, the main orientation estimates of CytoSpectre compare favorably against those of FibrilTool. The differences between the orientation errors produced by the two tools were statistically significant at the 1 % significance level in all cases except the non-degraded images of the cell cluster dataset. For the isotropy estimates, CytoSpectre obtained higher correlation coefficients than FibrilTool in all of the test cases for both datasets. However, the difference was less pronounced for the non-degraded and blurred cell cluster images.

**Table 3:** Performance comparison between CytoSpectre (CS) and FibrilTool (FT) using synthetic images of cell clusters

	CS orientation, error mean ± std (deg)	FT orientation, error mean ± std (deg)	Wilcoxon signed rank test p-value	CS circular variance, Pearson’s r	FT isotropy index, Pearson’s r
Non-degraded	1.7884 ± 3.6128	1.3904 ± 3.5042	0.0111	0.39844	0.31512
Blurred	1.8748 ± 3.9533	1.3432 ± 3.0325	0.0069	0.39645	0.31258
Gaussian noise	1.7101 ± 3.4064	6.4346 ± 10.067	4.0933E-14	0.38642	0.15947
Poisson noise	1.8325 ± 3.623	3.3052 ± 3.0916	1.0166E-08	0.38501	0.22644

Absolute errors in degrees (mean ± standard deviation) between true mean orientation values and values estimated by CS and FT are shown for images of the cell cluster dataset (*N* = 100) without degradations, with moderate blurring (kernel std 2.5 pixels), with moderate Gaussian noise (var. 1 %) and with moderate Poisson noise (level 10). Paired, two-sided Wilcoxon signed rank tests were performed to compare the orientation errors of CS and FT and the resulting p-values are given for each case. Pearson’s linear correlation coefficients between true and estimated measures of isotropy are also shown for the corresponding cases

**Table 4:** Performance comparison between CytoSpectre (CS) and FibrilTool (FT) using synthetic images of intracellular fibrils

	CS orientation, error mean ± std (deg)	FT orientation, error mean ± std (deg)	Wilcoxon signed rank test p-value	CS circular variance, Pearson’s r	FT isotropy index, Pearson’s r
Non-degraded	2.6261 ± 10.482	29.673 ± 39.693	5.6773E-12	0.54506	0.19542
Blurred	3.2969 ± 10.063	56.685 ± 38.136	2.6288E-22	0.15807	0.10137
Gaussian noise	2.2876 ± 10.11	15.785 ± 25.552	3.7340E-19	0.45714	0.2711
Poisson noise	3.7733 ± 13.852	19.829 ± 28.444	1.9903E-16	0.49203	0.22492

Absolute errors in degrees (mean ± standard deviation) between true mean orientation values and values estimated by CS and FT are shown for images of the fibril dataset (*N* = 100) without degradations, with moderate blurring (kernel std 2.5 pixels), with moderate Gaussian noise (var. 1 %) and with moderate Poisson noise (level 10). Paired, two-sided Wilcoxon signed rank tests were performed to compare the orientation errors of CS and FT and the resulting p-values are given for each case. Pearson’s linear correlation coefficients between true and estimated measures of isotropy are also shown for the corresponding cases

We also compared the performance of CytoSpectre and FibrilTool in the case of the three sets of real images described in the previous sections. The results already obtained by CytoSpectre in the validation experiments were used also for this comparison. Analysis of the real images using FibrilTool was performed similarly to the synthetic images, that is, by selecting the entire image as a ROI and by using the default line width settings. The adjustments applied to the orientations and anisotropy values estimated by FibrilTool were performed as described above for the synthetic images. Full FibrilTool outputs for the real images are provided in Additional file 7. As in the case of the validation experiments described in the previous sections, we compared the mean orientations and isotropy values estimated using FibrilTool to the mean orientations and anisotropy rankings performed by human experts for each validation set. We computed the mean and standard deviation of the absolute orientation errors over all experts (*N* = 12 for validation sets 1 and 2, *N* = 13 for validation set 3) and all images (*N* = 11 for validation set 1, *N* = 10 for validation sets 2 and 3) for each tool and each validation set. The number of images used for these evaluations is lower than the total number of images in each validation set (*N* = 15) due to the exclusion of highly isotropic images for which quantification of mean orientation is meaningless, as described in the previous sections. To evaluate the coherency of the computational and manual estimates of isotropy, Pearson’s linear correlation coefficients were calculated between the anisotropy rankings performed by each human expert and the circular variances and isotropy indices estimated by CytoSpectre and FibrilTool, respectively. The mean and standard deviation of the correlation coefficients obtained for each method were computed over all experts in order to compare the performance of the two tools. Finally, we used the paired, two-sided Wilcoxon signed rank test to assess the statistical significance of differences in the orientation errors and correlation coefficients obtained for CytoSpectre and FibrilTool. The results of this experiment are summarized in Table 5.

**Table 5:** Performance comparison between CytoSpectre (CS) and FibrilTool (FT) using real images

	CS orientation, error mean ± std (deg)	FT orientation, error mean ± std (deg)	Wilcoxon signed rank test p-value	CS circular variance, Pearson’s r, mean ± std	FT isotropy index, Pearson’s r, mean ± std	Wilcoxon signed rank test p-value
Validation set 1	15.5774 ± 14.5547	21.1937 ± 15.0628	2.2755E-11	0.3018 ± 0.4325	0.3024 ± 0.4251	0.8501
Validation set 2	11.2739 ± 11.9993	41.9884 ± 31.2416	1.0532E-15	0.3574 ± 0.3555	−0.0896 ± 0.1343	0.0122
Validation set 3	15.3819 ± 17.3992	16.0332 ± 14.1959	0.0016	0.7192 ± 0.1505	0.6086 ± 0.1440	7.3242E-04

Absolute errors in degrees (mean ± standard deviation) between mean orientation values estimated by human experts (*N* = 12 for validation sets 1 and 2, *N* = 13 for validation set 3) for the images of each validation set (*N* = 11 for validation set 1, *N* = 10 for validation sets 2 and 3) and values estimated by CS and FT are shown. Pearson’s linear correlation coefficients (mean ± standard deviation) between anisotropy rankings estimated by human experts (*N* = 12 for validation sets 1 and 2, *N* = 13 for validation set 3) and measures of isotropy estimated by CS and FT are also shown for the images of each set (*N* = 15 for all validation sets). Paired, two-sided Wilcoxon signed rank tests were performed to compare the orientation errors and correlation coefficients of CS and FT and the resulting p-values are given

For all three sets of real images, the mean errors between computational and manual estimates of mean orientation obtained for CytoSpectre were lower than the corresponding values of FibrilTool. The difference was statistically significant at the 1 % significance level in all three cases, but the most remarkable difference was observed in the case of validation set 2. For these images, the orientation error of CytoSpectre was approximately 11.3° ± 12.0° (mean ± standard deviation) while it was 42.0° ± 31.2° for FibrilTool. For validation set 1, the difference in the mean errors of the two tools was approximately five degrees, and less than one degree for the phase contrast micrographs of validation set 3. The mean linear correlation coefficients between the manual and computational measures of isotropy were almost identical for the two tools in the case of validation set 1. In the case of validation set 2, the mean correlation coefficient of CytoSpectre was considerably higher than that of FibrilTool, but the difference was not statistically significant at the 1 % significance level. In the case of validation set 3, CytoSpectre again outperformed FibrilTool and the difference reached statistical significance at the aforementioned significance level.

In summary, we found the accuracy of CytoSpectre to be comparable to that of FibrilTool for the cell cluster dataset without any added noise. However, even if the accuracy of the two methods is similar in this case, FibrilTool requires approximately 20 s of manual operation per ROI [1], which could mean minutes of labor for a single image in the case of multiple ROIs. In contrast, CytoSpectre only requires a few seconds of computation time per image, freeing the user for other tasks during an analysis run. In the presence of Gaussian or Poisson noise, CytoSpectre performed better than FibrilTool in terms of mean orientation error and linear correlation between the true circular variance and the estimated measure of isotropy. In the case of the fibril dataset, CytoSpectre outperformed FibrilTool in all cases. One of the main difficulties for FibrilTool in the images present in the fibril dataset is probably the shape of the fibril subunits, as they have variation in intensity also along the longitudinal orientation of the fibrils. The contributions from intensity gradients along the longitudinal and transverse orientations cannot be easily separated from each other in the spatial domain. Another issue present in many real images that is incorporated into the synthetic images of this dataset is the presence of interfering structures. The nucleus and the exterior of the cell’s cytoplasm also contribute to the orientation distribution estimated by FibrilTool and they too cannot be separated from the actual targets of interest in the spatial domain, unless some kind of segmentation steps are introduced. This separation is possible in the frequency domain, allowing CytoSpectre to obtain better results for such images. On the other hand, the cell cluster dataset does not feature such interfering structures and this advantage of spectral analysis does not bring any added benefit for CytoSpectre in that case. It is therefore not surprising, that the results produced by the two methods are very similar for that dataset, although CytoSpectre still appears to be less sensitive to noise.

The results obtained using real micrographs are rather similar to those based on synthetic images. From the viewpoint of statistical significance, the mean orientation estimates of CytoSpectre were clearly superior to those of FibrilTool for all three validation sets. In the case of validation set 1, CytoSpectre outperformed FibrilTool in terms of mean orientation error by several degrees. Validation set 2, consisting of images of hiPSC-CMs with their intracellular fibrils, proved to be very challenging for FibrilTool, resulting in a mean error over 30° higher than that of CytoSpectre. The difficulties encountered by FibrilTool when analyzing this set were probably due to the same reasons as those observed in the case of the synthetic fibril dataset, as discussed above. In the case of validation set 3, the magnitude of the difference, although in favor of CytoSpectre, is so small that it does not probably have any practical significance. This result resembles the case of the non-degraded and blurred images of the synthetic cell cluster dataset, in which case the small but statistically significant difference was in favor of FibrilTool. The synthetic images of the cell cluster dataset and the real images of validation set 3 both feature relatively homogeneous content, which appears to suit FibrilTool better than the images containing more distinct fibrillar structures. Results concerning the isotropy estimates produced by the two tools also varied from dataset to dataset. In the case of validation set 1, the performance of CytoSpectre was essentially identical to FibrilTool but for the other two datasets, CytoSpectre had a slight performance advantage, although the difference was not statistically significant in the case of validation set 2. Based on this experiment with real images, the results produced by CytoSpectre match subjective evaluations at least as well as those produced by FibrilTool, and in some cases CytoSpectre is clearly superior to FibrilTool. Of course, reliability of the subjective evaluations as a gold standard can be questioned in light of the considerable expert-to-expert variation observed in the validation experiments. The experiments based on synthetic images are not affected by this issue but they, on the other hand, cannot necessarily replicate all of the characteristics of real micrographs.

Finally, it should be noted that this comparison is not perfectly fair, since FibrilTool and CytoSpectre are based on quite different philosophies. FibrilTool allows (and requires) users to manually select ROIs and careful selection of these regions could probably improve the results when compared to our approach of selecting the entire image as a ROI. CytoSpectre, on the other hand, is meant to be used in automated fashion for analyzing large quantities of images with varying quality. This automated, high-throughput approach is reflected in the experimental setting of this comparative analysis, as manual selection of ROIs would have been unfeasible for such a large number of images and would have introduced human error into the results. With this in mind, we conclude that CytoSpectre appears to be superior to FibrilTool for such large-scale analyses. Still, the possibility of performing highly accurate measurements of mean orientation on carefully selected ROIs probably makes FibrilTool suited for particular types of experiments.

### Analysis of computational performance

In order to evaluate the computational performance of CytoSpectre, we benchmarked the software in terms of computation time required for images of different size. For this experiment, we used all of the real images (*N* = 15 per dataset) from validation sets 1, 2 and 3 as well as the first 15 images from each of the two synthetic image datasets. CytoSpectre’s default analysis settings were used for all datasets in this experiment. We used bilinear interpolation to scale the images to different sizes ranging from 500 × 500 pixels (0.25 megapixels) to 5000 × 5000 pixels (25 megapixels) before running the benchmarking experiment for each case. Benchmarking was performed using MATLAB’s *timeit* function, which runs the code multiple times for each input and stores the median time required for the computations. The analysis was performed using a 64-bit Windows 7 laptop PC equipped with an Intel Core™ i5 processor. The evaluation was applied to the main analysis function of CytoSpectre, which includes all of the actual processing and analysis steps performed for each image. The computation time required for updating the GUI is thus not included in these results. However, the GUI operates in essentially real time and the contribution of the computations required by the GUI on the overall computation time is negligible.

The computation times (mean ± standard deviation) in seconds for each dataset and image size are shown in Table 6. Some variation from dataset to dataset can be observed, but the computation time does not seem to depend heavily on the dataset, that is, on image content. As could be expected, the required computation time increases with increasing image size, but the increase is not dramatic for moderate image sizes (up to 9 megapixels). Based on these results, the computation times required for images of this size are typically 2–3 s. For the 16 megapixel and 25 megapixel images, the mean computation times were approximately 7–10 s. These results indicate that CytoSpectre can be used to study even relatively large datasets on basic hardware with acceptable computation times.

**Table 6:** Computation time per image for different datasets and image sizes

Image size (pixels)	Cell cluster dataset	Fibril dataset	Validation set 1	Validation set 2	Validation set 3
500 × 500	1.96 ± 0.46	1.93 ± 0.26	2.17 ± 0.32	2.18 ± 0.44	2.40 ± 0.39
1000 × 1000	1.91 ± 0.28	2.00 ± 0.30	2.26 ± 0.52	2.10 ± 0.38	2.62 ± 0.54
2000 × 2000	2.04 ± 0.18	2.10 ± 0.37	2.36 ± 0.34	2.17 ± 0.32	2.59 ± 0.61
3000 × 3000	2.24 ± 0.49	2.30 ± 0.32	2.40 ± 0.29	2.52 ± 0.42	2.62 ± 0.49
4000 × 4000	6.79 ± 0.73	7.40 ± 1.52	8.65 ± 1.52	7.84 ± 1.10	9.61 ± 2.06
5000 × 5000	7.05 ± 0.75	7.87 ± 1.85	8.36 ± 1.23	8.26 ± 1.63	9.77 ± 2.05

Computation time (mean ± standard deviation) in seconds required for analyzing a single image from each dataset at different image sizes. The analysis was performed using a laptop PC equipped with an Intel Core™ i5 processor

### Case study: effects of mechanical stretching on hiPSC-derived cardiomyocytes

To demonstrate the software in an actual use case, we applied CytoSpectre to a real experiment featuring mechanically loaded hiPSC-CMs. After Yamanaka’s Nobel winning breakthrough iPSCs have been widely used in disease modeling and drug screening [32–35]. Despite the advances in the field, cultured hiPSC-CMs are lacking in some functional and structural properties in vitro. In fact, they are considered to be immature, more fetal-like, than those found in adult tissue [36, 37]. Therefore, research on improving the maturity of these cells has become a hot topic lately [12, 13]. It has been shown that mechanical stress can promote the orientation and presumably also the maturation of hiPSC-CMs [38]. In this study, we investigated the effect of cyclic uniaxial mechanical stretching on hiPSC-CMs’ orientation with six different hiPSC lines. Generation and culturing of hiPSC lines, cardiac differentiation and mechanical loading with Flexcell® (Burlington, NC, USA) are described in detail in Additional file 4.

**Fig. 7:**
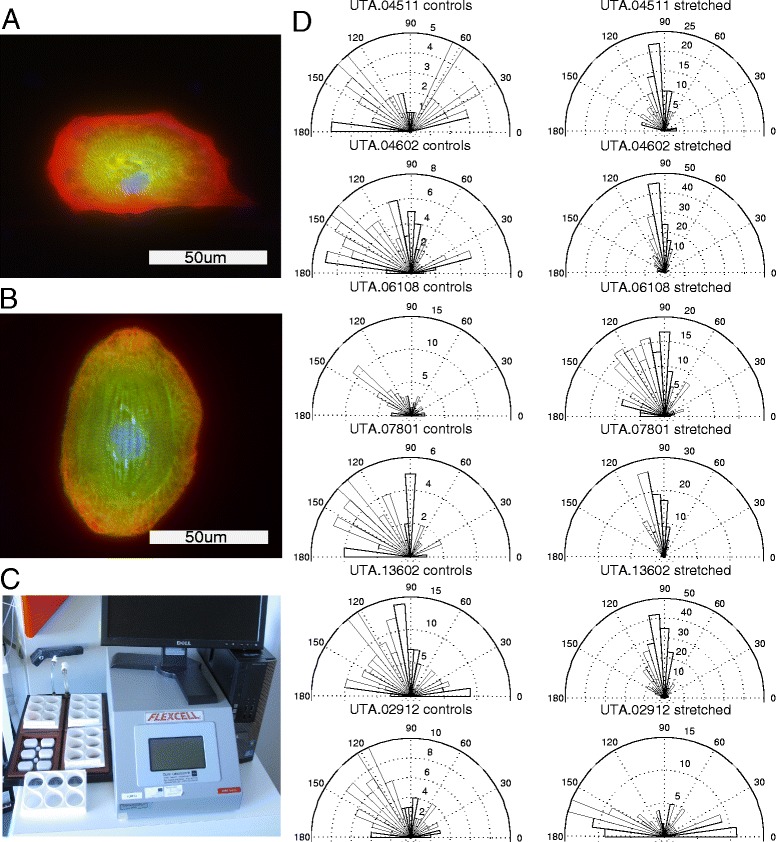
Quantitative analysis of the effect of mechanical stretching on the cellular orientation of hiPSC-CMs. Example images of a non-stretched control hiPSC-CM (**a**) and a stretched hiPSC-CM (**b**) are shown. The stretching experiments were performed using the FlexCell platform (**c**). Distributions of cellular mean orientations of control and stretched samples for different cell lines are shown in (**d**). The radial distance from the origin corresponds to the number of images whose mean orientations were within the limits of the corresponding orientation bin

CytoSpectre was used to analyze the mean orientations of cells in a large quantity of fluorescence microscopy images acquired from both control and stretched samples (see Fig. 7 for example images). Mixed component from the red channel, representing Alexa Fluor 568 secondary antibody against Troponin T or α-tropomyosin (both thin filament sarcomeric proteins), was analyzed from the images. Cells with circular variance exceeding 0.9 were excluded from the rest of the analysis, since mean orientation is not a meaningful quantity for highly isotropic distributions. Using the obtained mean orientation values describing the dominant orientation of each cell, we computed the mean orientation over all cells for each sample i.e., for each cell line and each culture condition (control vs. stretched). We also computed the circular variance of the cellular orientations for each sample. Watson-Williams tests were then performed to compare the control and stretched samples of each cell line. The Watson-Williams test is a circular analogue of the two sample *t*-test or the one-factor ANOVA and it can be used to assess the question whether the mean directions of two groups are identical or not. The calculations were performed using the CircStat toolbox [22]. The raw data and analysis settings can be found in the Additional file 9.

The results in Table 7 and Fig. 7 show that the mean orientation of the hiPSC-CMs subjected to stretching changes towards an axis that is perpendicular (orientation of 90°) to the axis of applied stress (horizontal i.e., orientation of 0° or 180°), when comparing to control samples. This result holds for five out of the six cell lines studied. In one cell line, UTA.02912, the observed change in mean orientation was opposite compared to the other lines. Also, we observed that circular variance decreased when comparing stretched samples to control samples. That is, cells subjected to stretching not only orientated towards a particular axis but their orientations were also concentrated more strongly along this particular orientation when compared to the more random orientation of cells in control samples. The results of the Watson-Williams tests indicate that the differences between the orientations of control and stretched samples are statistically significant at the 1 % significance level (*p* < 0.005) for each cell line. These results indicate that cyclic uniaxial stretching orientates the cells compared to static culture conditions. This finding could be relevant for tissue engineering when trying to create native, more mature, orientated myocardial tissue grafts in vitro.

**Table 7:** Summarized results of the mechanical stretching experiment

Cell line	Sample	N	Mean orientation	Circular variance	Watson-Williams test *p*-value
UTA.04511	Control	48	153°	0.92	0.00243
	Stretch	125	105°	0.46	
UTA.04602	Control	88	138°	0.68	1.76E-11
	Stretch	208	102°	0.31	
UTA.06108	Control	63	142°	0.63	5.18E-06
	Stretch	179	112°	0.56	
UTA.07801	Control	50	133°	0.57	1.05E-09
	Stretch	112	99°	0.21	
UTA.13602	Control	161	127°	0.72	1.77E-08
	Stretch	254	103°	0.34	
UTA.02912	Control	93	128°	0.69	5.72E-10
	Stretch	112	171°	0.60	

Cellular mean orientations, circular variance and Watson-Williams test p-values for different cell lines cultured under control vs. stretching conditions are shown. *N* = number of images analyzed per sample. The orientation of stretching was 0°, or equivalently, 180°

## Conclusions

We developed CytoSpectre, a software tool implemented as a standalone MATLAB application, allowing spectral analysis of microscopy images. The tool can be operated via a GUI on basic hardware without prior experience of programming or image processing. Spectral analysis allows users to extract information about the orientation and size distributions of targets within the images. In addition to analyzing complete images, the analysis can be targeted to features of a particular size range in order to obtain information only on these targets of interest while excluding other structures present in the image. Analyses can be performed automatically for large quantities of images and the results can be exported to spreadsheets and text files or plotted as images for further study. We analyzed the performance of the software by extensive simulations using computer-generated images with varying types and levels of image quality degradations and observed high tolerance against realistic amounts of blurring and Gaussian or Poisson distributed noise. The performance of CytoSpectre in the case of real images was validated using fluorescence microscopy images of hiPSC-CMs and phase contrast microscopy images of hiPSC-PSNs. Comparison with manual measurements performed for these images by a large panel of human experts showed that CytoSpectre is not limited to a single microscopy technique or cell type and is able to produce results which are in agreement with the majority of human experts. Furthermore, we observed a great deal of variation from expert to expert, underlining the need for automated computational tools such as CytoSpectre. We benchmarked CytoSpectre against FibrilTool [1], a state-of-the-art orientation analysis tool, using both synthetic and real images. In the case of synthetic images featuring simple targets without any degradation in image quality, CytoSpectre offered accuracy comparable to that of FibrilTool with the important added benefit of rapid, fully automated operation. For synthetic images with degraded quality or more realistic targets, CytoSpectre outperformed FibrilTool also in terms of accuracy. In the case of real images, CytoSpectre produced mean orientation estimates with accuracy superior to those of FibrilTool. The accuracy of estimated isotropy measures was either superior to or on a par with those of FibrilTool, depending on dataset. Finally, we demonstrated the software in a real experiment by analyzing images of hiPSC-CMs subjected to cyclic uniaxial stretching. The results of this experiment suggest that stretched hiPSC-CMs orientate along an axis that is transverse to the orientation of stretching, a finding which could have relevance for tissue engineering applications. We hope CytoSpectre will be useful in various applications where the orientations and/or size distributions of biological structures are of interest. The main benefits offered by the software are the high level of automation, ease of use, versatility in terms of applications and good tolerance against degraded image quality. Since the software is compatible with any kind of 2D images, it could also be useful in biology related or even non-biological applications benefiting from spectral analysis such as (bio)materials research [9, 39] or geophysics [40].

### Availability and requirements

**Project name:** CytoSpectre

**Project home page:**http://www.tut.fi/cytospectre

**Operating system(s):** Windows 7 64-bit (standalone deployed application)

**Programming language:** MATLAB (R2014a)

**Other requirements:** For standalone operation on Windows 7 (64-bit) systems without a MATLAB installation, MATLAB Compiler Runtime (version 8.3) is required. The MATLAB Compiler Runtime can be automatically obtained during installation. Operating the software in source code form via MATLAB requires the following toolboxes: Signal Processing Toolbox, Image Processing Toolbox, Statistics Toolbox and Curve Fitting Toolbox.

**License:** All original CytoSpectre source codes are governed by the GNU General Public License (GPL). CircStat toolbox source codes are governed by the corresponding CircStat license. The standalone deployed application is governed by the MATLAB Compiler Runtime license.

**Any restrictions to use by non-academics:** The standalone deployed application may only be used for the sole purpose of academic research in accordance with the MATLAB Compiler Runtime license. No restrictions apply for the software in MATLAB source code form.

### Ethics statement

The collection of biopsies for generating patient specific hiPSC lines was approved by the ethical committee of Pirkanmaa Hospital District (Aalto-Setälä R08070). Written informed consent was obtained from all the donors.

## Additional files

Additional file 1:
**CytoSpectre installer.** This file contains the installation package for the Windows 7 (64-bit) deployed application. (EXE 10939 kb) Additional file 2:
**CytoSpectre source code.** This file contains the MATLAB (R2014a) source codes. (7Z 78 kb) Additional file 3:
**CytoSpectre user guide.** This file contains the user guide of the software. (PDF 464 kb) Additional file 4:
**Supplementary information.** This file contains detailed descriptions of computational and experimental methods as well as results and discussion of the sensitivity analysis. (PDF 715 kb) Additional file 5:
**Raw data from sensitivity experiments.** This file contains all numerical data from the experiments evaluating the sensitivity of the results to changes in the values of adjustable parameters. (XLSX 8008 kb) Additional file 6:
**Raw data from image degradation experiments.** This file contains all numerical data from the image quality degradation experiments. (XLSX 19997 kb) Additional file 7:
**Raw data from experiments with real images.** This file contains all numerical data from the experiments with real images. (XLSX 45 kb) Additional file 8:
**Raw data from FibrilTool experiments with synthetic images.** This file contains all numerical data from the comparative experiments with FibrilTool using synthetic images. (XLSX 336 kb) Additional file 9:
**Raw data from the case study.** This file contains all numerical data from the case study experiment. (XLSX 919 kb)

## Abbreviations

PSD: Power spectral density
GUI: Graphical user interface
hiPSC: Human induced pluripotent stem cell
hiPSC-CM: Human induced pluripotent stem cell derived cardiomyocyte
hiPSC-PSN: Human induced pluripotent stem cell derived peripheral sensory neuron
WOSA: Weighted overlapped segment averaging
DFT: Discrete Fourier transform
ROI: Region of interest
CS: CytoSpectre
FT: FibrilTool
