# Supplementary material for: CytoSpectre: a tool for spectral analysis of oriented structures on cellular and subcellular levels
Source: BMC Bioinformatics. 2015 Oct 26;16:344. doi: 10.1186/s12859-015-0782-y (PMC4624586; doi:10.1186/s12859-015-0782-y)
Supplement: Additional file 3: — CytoSpectre user guide. This file contains the user guide of the software. (PDF 464 kb) [file 12859_2015_782_MOESM3_ESM.pdf]

CytoSpectre User Guide

Version 1.1, August 27, 2015

K. Kartasalo *et al.*, CytoSpectre: a tool for spectral analysis of oriented structures on cellular and subcellular levels, 2015.

http://www.tut.fi/cytospectre

BioMediTech – Institute of Biosciences and Medical Technology, Tampere, Finland
Tampere University of Technology, Tampere, Finland
University of Tampere, Tampere, Finland

Contents

1 Introduction 3

2 Understanding spectral analysis 3

3 Installing the software 3

4 Quick start guide 4

5 Settings and profiles 6

5.1 General settings 6

5.2 Analysis settings 6

5.3 Saving/Loading profiles 11

6 Running an analysis 12

6.1 Importing images 12

6.2 Analyzing images one by one 12

6.3 Analyzing images in batch mode 12

7 Inspecting results 13

7.1 General information 13

7.2 Morphological statistics 14

7.3 Orientation statistics 15

7.4 Wavelength statistics 16

7.5 Detail component signal-to-noise ratio 16

7.6 Power spectrum 17

8 Exporting results 18
8.1 Exporting to spreadsheets or text 18
8.2 Exporting raw data 18
8.3 Exporting plots as images 19
9 Operation via the MATLAB command line 20
10 Troubleshooting 21
11 License agreements 23
11.1 MATLAB source code form 23
11.2 Deployed application 24

1 Introduction

CytoSpectre is a software tool for the analysis of orientation and wavelength distributions from micrographs. It is based on spectral analysis and allows analyzing various structures of different dimensions e.g. clusters of cells or intracellular fibrillar assemblies. In principle, CytoSpectre is compatible with 2D images obtained using any form of microscopy. In addition to quantifying the mean orientation of the structures of interest, estimates of parameters describing the anisotropy and shape of the distributions can also be obtained. A similar analysis can be performed for wavelength distributions, which describe the typical dimensions of the target structures.

CytoSpectre is implemented in MATLAB, but a standalone deployed application for Windows is also freely available for academic use. The software can be operated on basic desktop computers via a graphical user interface (GUI). Analysis results can be easily exported to spreadsheets or text files for further study or plotted as images for visualization purposes.

2 Understanding spectral analysis

Instead of analyzing images as such (i.e. in their original spatial form), spectral analysis works by transforming images into a frequency representation by decomposing them into a spectrum of periodic components. That is, structures having different characteristic dimensions and different orientations are located at different regions of the spectrum. For example, the spectrum computed from a high magnification image of a cell might contain low frequency (i.e. high wavelength) contributions from relatively large structures such as the overall shape of the cell. Finer details, such as intracellular fibrils or organelles, would be manifested as high frequency (i.e. short wavelength) signals in the spectrum. By analyzing the spectrum, one can estimate both orientation and spatial frequency or wavelength distributions of the different structures present in the image. In addition to analyzing the whole spectrum and presenting the results in a ‘mixed component’, CytoSpectre can also detect a ‘detail component’, which represents a separate part of the power spectrum of the image. This component can be used to study particular structures of interest, if they are present at a certain range of length scales and/or orientations. As an example of such structures, we have studied the sarcomeres of cardiac myocytes, as the subunits of these fibrillar structures have a relatively constant characteristic length of two micrometers. The method is described in more detail in the corresponding article.

3 Installing the software

CytoSpectre can be downloaded from http://www.tut.fi/cytospectre. Users without a MATLAB installation can download the deployed application, which can be operated without a MATLAB installation. The deployed application is compatible with 64-bit Windows systems (tested with Windows 7) and requires the installation of MATLAB Compiler Runtime (MCR) libraries (version 8.3) in order to work. The MCR package can be downloaded bundled as part of the CytoSpectre installation package or it can be obtained automatically during CytoSpectre installation via an internet connection. In either case, the MCR libraries will be automatically

installed during CytoSpectre installation. Please note that administrator privileges are required during installation. While CytoSpectre can be installed and used for free, users of the deployed application must agree to follow the license agreements governing the MCR libraries and deployed MATLAB applications. Most importantly, only academic research use of the CytoSpectre deployed application is permitted. Use for other purposes is forbidden. For details, see Section 11. The deployed application can be operated via a graphical user interface (GUI).

Users with a MATLAB installation can download the MATLAB source codes (R2014a) of CytoSpectre and run the software either via the GUI or via the MATLAB command line. Otherwise the source code form of the software is identical with the deployed application. The source codes, except for the CircStat toolbox[1] functions, are licensed under the GNU General Public License (GPL). The CircStat toolbox by Philipp Berens is governed by a separate license. See Section 11 for details.

For convenient operation, we recommend using hardware with sufficient computational power for image processing, e.g. at least an Intel CORE™ i5 processor or equivalent. In terms of memory, CytoSpectre does not have special requirements for most images, as only one image is loaded into memory at a time.

4 Quick start guide

This section gives brief step-by-step instructions for performing a simple analysis using default settings. A basic analysis can be performed automatically in a matter of minutes. Please note that while the default settings used here produce satisfactory results for many typical images, they may not be suitable for your application. For detailed explanations of the various settings and features of CytoSpectre, see the following Sections 5-8 and the troubleshooting tips of Section 10.

1. If you are using the deployed application, start the software by double-clicking on the **CytoSpectre** icon. If you are running the software via MATLAB, navigate to the folder containing the source code files and run **CytoSpectreGUI.m**.
2. Add images by clicking **Add** and browsing for your image files. Once you have selected the images, click **OK**.
3. Click **Preferences -> Analysis settings** to open the analysis settings window.
4. Insert the **Magnification** of your images in the box in the upper left corner, e.g. “20”. Please remember that you should insert the combined magnification of the objective and any camera adapter used to connect the camera to the microscope.
5. Insert the pixel size of your camera in micrometers in the **Camera pixel size** box, e.g. “7.4”. This information can be found in the technical specifications of the camera, which can usually be easily obtained from the camera manufacturer’s website. If you are using binning i.e. averaging multiple adjacent pixels, this has to be taken into account.

[1] P. Berens, CircStat: A Matlab Toolbox for Circular Statistics, Journal of Statistical Software, 2009. http://bethgelab.org/software/circstat/

6. Select the **Target channel** i.e. the color channel (red, green, blue) you wish to analyze from a color image. Selecting "Combined" will merge all color channels into one. For grayscale images, the target channel setting has no effect and it can be ignored. Click **OK**.
7. Click **Analyze all images** and wait until the analysis is finished. This can take several minutes, depending on the number and size of images.
8. Inspect the orientation distribution plot at the left side of the bottom panel. The plot depicts the distribution of oriented structures in the image. Summary statistics at the right side of the bottom panel include the mean direction i.e. the most common orientation in the image and circular variance, which is a measure of the isotropy of the orientation distribution. A circular variance of zero corresponds to perfect alignment of all oriented structures along a single line. A circular variance of one, on the other hand, corresponds to perfect lacking of a dominant orientation.
9. Inspect the wavelength distribution plot at the right side of the bottom panel. The plot depicts the distribution of wavelengths in the image. Wavelengths can be interpreted as measures of the characteristic dimensions of structures in the image. Small, densely packed objects are represented by short wavelengths while large structures are represented by long wavelengths. Summary statistics at the right side of the bottom panel include the mean, median and mode of the wavelengths, as well variance and standard deviation, which are measures of the spread of the wavelength distribution.
10. Switch between the **Mixed component** and the **Detail component** by clicking the corresponding buttons in the **Select component** section at the left side of the bottom panel. The orientation and wavelength distributions and statistics are shown for the selected component. The mixed component represents all structures within the image while the detail component attempts to capture particular target structures within the image.
11. Open the result export window by clicking **Export results**. Select **All images**. Select **Spreadsheet summary** for a Microsoft Excel compatible spreadsheet file format, **Text summary** for a general text file format or **Figures** for plots. Click **Export**, select a filename for saving your data and click **OK**.

5 Settings and profiles

5.1 General settings

General settings can be accessed by clicking **Preferences -> General settings**.

Setting	Description	Options
Location of temporary files	Temporary files are created for each image during an analysis and automatically deleted when no longer needed. These files can be saved at the same locations as the input images or at a custom location specified by the user. Depending on the analysis settings used, the temporary files require approximately 1-5 times the disk space of the original images.	Same as images (default)<br>Custom location
Length units	The length unit used for all wavelength values and morphological data can be selected to suit the dimensions of the target.	fm<br>pm<br>nm<br>µm (default)<br>mm<br>cm<br>m<br>km

Note: *General settings cannot be modified during an analysis session i.e. when images have already been imported for analysis. Modifying general settings requires clearing the current session.*

5.2 Analysis settings

Analysis settings can be accessed by clicking **Preferences -> Analysis settings**.

The following settings control the basic spectral analysis procedure.

Setting	Description	Options
Magnification	Magnification of the images can be set as fixed, e.g. “20”, or detected automatically from the filename of each image.<br><br>Detection from filename requires the filename to include a substring with the magnification value preceded or	Fixed (default, 40)<br>Detect from filename

	followed by an X (case-insensitive), e.g. X20 or 20X. The magnification value can have a decimal separator (period, not comma), e.g. 20.5X. The substring can be located at any position in the filename but the X must be preceded or followed by a non-numeric, non-alphabetic character. For example, the filenames “myImage20x.tif” and “20x-myImage.tif” are valid, but “myImagex20.tif” and “20x5678.tif” will not be correctly recognized.<br><br>Note that some camera adapters affect the magnification. For example, a 10X objective and a 0.63X camera adapter would yield a combined magnification of 6.3X.	
Camera pixel size	The physical pixel size of the camera sensor in micrometers, e.g. “7.4”.<br><br>This information can be found in the technical specifications of the camera, which can usually be easily obtained from the camera manufacturer’s website.<br><br>Note that if you are using binning i.e. averaging multiple adjacent pixels, this has to be taken into account. For example, for a camera with a pixel size of 7.4 µm x 7.4 µm, 2x2 binning would result in an effective pixel size of 14.8 µm x 14.8 µm.	Default: 6.8
Import cell segmentation	By default, the whole image is analyzed and the analysis results represent all structures within the image. If cell segmentation (i.e. partition of the image into cells and background) is available, it can be imported into CytoSpectre, allowing cell-by-cell analysis.	Disabled (default)<br>Enabled

	Segmentation can be performed with many publicly available algorithms[2] or it can be done manually.<br><br>If segmentation is enabled, a mask image named segcell_<imagename> e.g. "segcell_myImage.tif" has to be placed in the same directory as the corresponding actual image. The mask can be a binary image which has zero intensity (black) at pixels belonging to background and high intensity (white) at pixels belonging to cells. Each connected high intensity region is then assumed to be a single cell. Alternatively, the mask can be a label image, where background pixels have the value zero, pixels corresponding to the first cell have value 1, pixels corresponding to the second cell have value 2 and so on.	
Target channel	The color channel to analyze from a color image. Selecting "Combined" will merge all color channels into one. For grayscale images, the target channel setting has no effect and it can be ignored.	Red<br>Green (default)<br>Blue<br>Combined
Rotate orientation by 90 degrees	Spectral analysis captures the orientation of intensity variations in an image. Typically this orientation is transverse relative to the orientation of the structures of interest and the orientations calculated from the spectrum thus have to be rotated by 90 degrees. In these cases, the rotation setting should be enabled.<br><br>In some cases, intensity variation occurs parallel to the orientation of the structures of interest. For example, myofibrils exhibit a striated intensity pattern along the longitudinal direction of the fibrils. In such cases, the rotation	Disabled<br>Enabled (default)

[2]Examples of popular tools with cell segmentation functionality include CellProfiler (http://www.cellprofiler.org/) and ImageJ (http://imagej.nih.gov/ij/).

	correction should be disabled.<br><br>The correction can be applied separately to the mixed and detail components. This may be useful e.g. when most of the structures in the image are typical ones with transverse intensity variation but specific structures of interest exhibit longitudinal intensity variation. In such a situation, the mixed component rotation can be enabled and the detail component rotation disabled.	
Spectral resolution/noise	During spectral estimation, a trade-off between spectral resolution and noise has to be made. High resolution allows more subtle details of the orientation and wavelength distributions to be seen, but this comes at the expense of increased noise i.e. random fluctuations in the spectrum and the distributions extracted from it.	Maximum resolution<br>High resolution<br>Balanced (default)<br>Low noise<br>Minimum noise
Minimum wavelength	By default, the minimum wavelength analyzed is limited only by the resolution of the imaging system. A manual minimum wavelength limit can be set to exclude structures whose characteristic dimensions are smaller than this limit. This can be useful e.g. in order to reduce the effects of interfering low wavelength patterns such as grooves on a cell culture substrate or artefacts produced by the camera sensor. The manual minimum wavelength is given in the units specified by the **Length units** setting.	Unlimited (default)<br>Manual
Maximum wavelength	By default, the maximum wavelength analyzed is set automatically to only remove long wavelength artefacts caused by the spectral estimation process. A manual maximum wavelength limit can be set to exclude structures whose characteristic dimensions are larger than this limit. This can be useful e.g. if the user is only interested in finer details of the image. The manual maximum wavelength is	Automatic (default)<br>Manual

	given in the units specified by the **Length units** setting.	
Exclude wavelength band	By default, the whole spectrum between the minimum and maximum wavelengths is analyzed. A given band of wavelengths can also be excluded from the analysis by enabling this setting. This can be useful e.g. if there are interfering patterns or artefacts whose characteristic dimensions are known to be within a certain range of values. The minimum and maximum wavelengths to exclude are given in the units specified by the **Length units** setting.	Disabled (default)<br>Enabled
Detail component wavelength band	By default, the detail component is extracted automatically based on signal-to-noise ratios. Semi-automatic detection can be used if the characteristic dimensions of the structures of interest are known or a reasonable guess is available. In this case, the detail component is automatically extracted based on the given range of expected wavelengths.<br><br>For example, sarcomeric subunit-to-subunit distance along cardiac myofibrils is well known to be approximately 2 µm in normal cardiac myocytes. In order to detect these structures, the semi-automatic setting and a wavelength range of e.g. 1.5 µm – 2.5 µm could be used. The minimum and maximum wavelengths of the detail component wavelength band are given in the units specified by the **Length units** setting.	Automatic (default)<br>Semi-automatic

The following advanced settings control the detail component detection process and do not typically need to be modified from their default values.

**Setting**	**Description**	**Options**
Segment length	Length of data segment used during background spectrum estimation and detection of main directions. Large	Default: 0.1

	segment length produces very smooth background spectra but may fail to follow the real shape of the background spectrum. Small segment length produces spikier background estimates which may include significant contributions from the actual signal. Size of the segment is given relative to the total number of frequency bins in the spectrum. Segment length should be set between 0 and 1.	
Significance level	Significance level used during main direction estimation. Statistical testing is performed to detect a transition between low and high spatial frequency ranges of the spectrum and the selected significance level is used for the test. Significance level should be set to a value larger than zero but less than one. Typical choice is 0.05 or 0.01.	Default: 0.05
Angular tolerance	Range of orientation angles used around the main orientation during detail component detection. Given in degrees.	Default: 10
Convergence limit	Convergence threshold for iterative fine-tuning of the detail component spectral region. Iterations are stopped when both the mean direction and the angular standard deviation of the detail component change by less than the threshold value between successive iterations. Given in degrees.	Default: 1
Max. iterations	Maximum number of iterations for iterative fine-tuning of the detail component spectral region.	Default: 10
Prior width, angle	Multiplier used for the initial width of the expected detail component spectral region along the angle axis.	Default: 0.5

5.3 Saving/Loading profiles

User profiles can be saved by clicking **File -> Save profile** and inputting a name for the profile. Profiles can be loaded by clicking **File -> Load profile** and browsing for an existing profile file. A user profile contains the general settings and analysis settings, allowing users to save and retrieve settings that have been found suitable for a particular cell type, microscopy technique etc.

6 Running an analysis

6.1 Importing images

Images can be imported for analysis by clicking **Add** and browsing for the images of interest. Note that you can select multiple images by holding down Ctrl or Shift. Once all the images have been selected, click **OK**. You can now use the menu on the left hand side of the GUI to select which image to view. Use the magnifier and pan tools at the upper left corner to zoom in and out and move the view within the image. The data cursor tool can be used to inspect intensity values.

Imported images can be removed from CytoSpectre by selecting the image from the menu and clicking **Remove**. If you wish to remove all of the imported images, click **Clear all**.

Note: *CytoSpectre is compatible with .tif, .jpeg, .gif., .bmp and .png image formats. Images can be either grayscale or RGB. CytoSpectre only supports 2D images but z-stacks can be analyzed image by image. There is no limit on image size, but computation time is increased for images with a larger number of pixels.*

6.2 Analyzing images one by one

To analyze the currently selected image, click **Analyze this image** at the lower right corner. The analysis may take a moment to start, especially for large images. Analyzing images one by one is useful especially for troubleshooting or testing of different settings.

6.3 Analyzing images in batch mode

To analyze all of the imported images, click **Analyze all images** at the lower right corner. A dialog will pop up, asking if you wish to skip previously analyzed images or analyze all images, overwriting any previous results. If you wish to only analyze images not already analyzed, click **Skip analyzed images**. If you wish to (re)analyze all images, click **Analyze all**. Skipping previously analyzed images can be useful if you have images requiring different magnification settings, for example. In that case, you can first import and analyze one batch of images. You can then import another batch of images, change the settings accordingly, and analyze these images while retaining the results already computed for the first batch.

Depending on the analysis settings and image size, analyzing a single image without segmentation can typically take 3-10 seconds on an Intel CORE™ i5 or similar processor. Large images take longer to analyze than smaller images. See Section 10 for tips on reducing the time required for computation. The analysis run can be aborted at any time by clicking **Abort**. The analysis will then be stopped after the current image.

7 Inspecting results

7.1 General information

General information about the selected image and the settings used during analysis are shown in the panel at the top right corner of the CytoSpectre GUI. Values given in <length units> refer to the physical length unit specified by the **Length units** setting (see Section 5.1.). For more information about the analysis settings listed here, see Section 5.2.

Filename: Full path and name of the image file.

Image size (pixels): Size of the image in pixels (width x height).

Image size (<length units>): Size of the image in physical length units (width x height).

Magnification: Magnification set by the user or detected from the filename.

Camera pixel size (µm): Camera pixel size specified by the user, in micrometers.

Image pixel size (<length units>): Corresponding physical size of image pixels, calculated as camera pixel size divided by magnification.

Segmentation: Segmentation enabled/disabled.

Target channel: Analyzed color channel.

Resolution/noise: Spectral resolution/noise setting.

Rotate mixed: Rotation correction for mixed component enabled/disabled.

Rotate detail: Rotation correction for detail component enabled/disabled.

Min wavelength (<length units>): Actual minimum wavelength/minimum wavelength specified by the user. The actual value can differ from the user specified value e.g. if the value specified by the user is lower than the minimum wavelength dictated by the resolution of the imaging system.

Max wavelength (<length units>): Actual maximum wavelength/maximum wavelength specified by the user. The actual value can differ from the user specified value e.g. if the value specified by the user is higher than the maximum wavelength dictated by the physical distance covered by the image.

Excluded, min (<length units>): Actual shortest wavelength excluded/shortest wavelength to exclude specified by the user. The actual value can differ from the user specified value e.g. if the value specified by the user is not within minimum and maximum wavelengths dictated by the imaging system.

Excluded, max (<length units>): Actual longest wavelength excluded/longest wavelength to exclude specified by the user. The actual value can differ from the user specified value e.g. if the value specified by the user is not within minimum and maximum wavelengths dictated by the imaging system.

Detail guess, min (<length units>): Actual expected minimum wavelength for the detail component/expected minimum wavelength for the detail component as specified by the user. The actual value can differ from the user specified value e.g. if the value specified by the user is not within minimum and maximum wavelengths dictated by the imaging system.

Detail guess, max (<length units>): Actual expected maximum wavelength for the detail component/expected maximum wavelength for the detail component as specified by the user. The actual value can differ from the user specified value e.g. if the value specified by the user is not within minimum and maximum wavelengths dictated by the imaging system.

Detail search settings: Advanced search settings used for detail component detection, default or custom. Used values for the parameters listed as: [**Segment length**; **Significance level; Angular tolerance; Convergence limit; Max. iterations; Prior width**].

7.2 Morphological statistics

If segmentation is enabled and a mask image is found (see Section 5.2. for more information), several measures of cellular morphology are calculated and shown for each cell in the morphological data panel. Values given in <length units> refer to the physical length unit specified by the **Length units** setting (see Section 5.1.). Cells are numbered with numerical indices starting from one. Individual cells can be selected by clicking on the cell indices in the list left of the morphological data panel. The outlines of the selected cell are overlaid on the image in red. The morphological statistics include:

Center X (pixels): The x-coordinate of the cell's centroid in pixels (starting from left).

Center Y (pixels): The y-coordinate of the cell's centroid in pixels (starting from top).

Center X (<length units>): The physical x-coordinate of the cell's centroid (starting from left).

Center Y (<length units>): The physical y-coordinate of the cell's centroid (starting from top).

Area (<length units>^{2}): The physical area covered by the cell.

Perimeter (<length units>): The physical perimeter of the cell i.e. the distance along the outline of the cell.

Major axis (<length units>): The length of the major axis of an ellipse that has the same normalized second central moments as the cell region i.e. the 'length' of the cell along its longitudinal axis.

Minor axis (<length units>): The length of the minor axis of an ellipse that has the same normalized second central moments as the cell region i.e. the 'width' of the cell along its transverse axis.

Eccentricity: The ratio of the distance between the foci of an ellipse that has the same normalized second central moments as the cell region and the major axis length of the ellipse. Eccentricity values lie in the interval [0, 1] with a value of zero corresponding to a circle and a value of one corresponding to a line segment. In other words, cells that have an elongated shape have a higher eccentricity than cells whose shape is round.

Solidity: The ratio of cell area and the area of the cell's convex hull. Solidity values lie in the interval (0, 1] with smoothly shaped cells having a high value and cells with protrusions having a low value.

7.3 Orientation statistics

The distribution of orientations for the selected cell in the selected image is shown as a plot in the spectral analysis results panel. You can switch between the mixed and detail components by clicking on the **Mixed component** and **Detail component** radio buttons on the left hand side of the panel. The distance from origin represents the relative frequency of the corresponding orientation. The following summary statistics are shown at the top right corner of the panel:

Mean orientation: The dominant orientation in degrees. Note that orientations repeat every 180 degrees i.e. the mean orientation always lies in the interval [0, 180) degrees.

Circular variance: A measure of spread of the orientation distribution. Circular variance is bounded in the interval [0, 1]. In the case of perfect anisotropy i.e. the whole distribution aligned along a single orientation, circular variance has a value close to zero. In the case of perfect isotropy i.e. the whole distribution spread out evenly around the half circle, circular variance has a value close to one. Note that high circular variance does not imply a uniform distribution but only the lack of any dominant direction.

Angular standard deviation: An alternative measure for the spread of the orientation distribution. Angular standard deviation is bounded in the interval $[0, \sqrt{2}]$. Similarly to circular variance, low values indicate anisotropy while high values indicate isotropy of the distribution.

Circular skewness: A measure of the symmetry of the distribution. Values close to 0 are indicative of a distribution that is symmetric around the mean orientation.

Circular kurtosis: A measure of peakedness. Large positive values indicate a strongly peaked distribution i.e. a distribution whose values are mostly concentrated to a limited number of orientation angles. Low values indicate a distribution whose values are 'smoothly' distributed across many different orientation angles. Note that circular kurtosis is different from the measures of circular spread (i.e. circular variance, circular standard deviation and angular standard deviation). A high circular kurtosis value does not imply the presence of a dominant mean orientation or necessarily coincide with low values of the measures of circular spread.

Even if the value of circular kurtosis is high and the distribution is thus concentrated to a small number of orientation angles, these angles may be spread out evenly around the half-circle, resulting in high values for the measures of circular spread.

***Note:** CytoSpectre uses circular statistics to handle orientation distributions. Circular statistics is a subfield of statistics used for data defined on scales lacking a designated zero, such as orientation data. For example, the mean of several orientation values cannot be correctly calculated with the common formula of the arithmetic mean intended for data on a linear scale. If you are performing further calculations with the mean directions computed by CytoSpectre, remember to use techniques appropriate for circular data. Apart from the mean direction, other summary statistics computed by CytoSpectre are defined on a linear scale, even if the data they describe is circular. Statistical testing or other further analysis can therefore be performed with conventional techniques for these statistics. More information about circular statistics can be obtained e.g. from the CircStat paper[3] or books on circular statistics[4].*

7.4 Wavelength statistics

The distribution of wavelengths for the selected cell in the selected image is shown as a plot in the spectral analysis results panel. You can switch between the mixed and detail components by clicking on the **Mixed component** and **Detail component** radio buttons on the left hand side of the panel. The x-axis indicates wavelength values, given in the physical length units specified by the **Length units** setting (see Section 5.1.). The y-axis indicates the relative frequency of each wavelength. The following summary statistics are shown at the bottom right corner of the panel, with mean, median, mode, variance and standard deviation also given in the specified length units:

Mean wavelength: The mean of the wavelength distribution.

Median wavelength: The median of the wavelength distribution.

Mode: The mode of the wavelength distribution.

Standard deviation: The standard deviation of the wavelength distribution is a measure of the spread of the wavelength values. Standard deviation is the square root of variance.

7.5 Detail component signal-to-noise ratio

The signal-to-noise ratio (SNR) of the detected detail component is shown below the component selection radio buttons. SNR is a measure of signal power relative to an estimated background spectrum and it is given in decibels. A high SNR indicates that the detected detail component represents some kind of signal rather than random noise. However, this does not guarantee that the signal is representative of any structures of interest. Background patterns in the image (such

[3] P. Berens, CircStat: A Matlab Toolbox for Circular Statistics, Journal of Statistical Software, 2009.

[4] N. I. Fisher, Statistical Analysis of Circular Data, Cambridge University Press, 1995.; K. V. Mardia, P. E. Jupp, Directional Statistics, Wiley, 1999.

as vertical or horizontal periodically repeating lines) caused by camera electronics or incorrect calibration, for example, can create ‘signals’ with high SNR values. An SNR of zero decibels means that the power of the ‘signals’ captured in the detail component equals the power of the estimated background, indicating that the detail component does not represent anything else than background noise. Please note that the decibel scale is logarithmic: an increase of 3 dB approximately corresponds to doubling of signal power. The minimum SNR which should be accepted generally depends on the types of images and the application and it is therefore difficult to give a generally applicable numerical threshold. We suggest that the SNR values are used as a supporting measure in combination with visual assessment of image quality. Images exhibiting low SNR values relative to similar types of images should be visually examined to evaluate if they are of sufficient quality and if the target structures are clearly visible or not.

7.6 Power spectrum

The power spectrum of the selected cell in the selected image can be inspected by clicking on the **View power spectrum** button. The power spectrum is shown in a new window. Use the magnifier and pan tools at the upper left corner to zoom and move around the spectrum. The data cursor tool can be used to inspect the values of the spectrum.

The power spectrum is shown in polar coordinates with orientation angles on the y-axis and spatial frequencies on the x-axis. The angle convention is similar to the orientation plot (see Section 7.3.) but orientations shown in the power spectrum typically differ by 90 degrees when compared to the orientations of the structures of interest (see the explanation of the “Rotate orientation by 90 degrees” setting in Section 5.2.). Spatial frequencies are given according to the physical length units specified by the **Length units** setting (see Section 5.1.). The values of the power spectrum at different orientations and spatial frequencies are given on a decibel scale (i.e. logarithmic scale of base 10) to make the typically faint high frequency signals visible. The power spectrum plot also shows information about the mixed and detail components. The spatial frequencies corresponding to the maximum and minimum wavelength limits (see Section 5.2.) used in the analysis are visualized as black outlines. The region of the spectrum detected as the detail component is outlined in red. The number of iterations performed while detecting the detail component is also shown above the plot.

Spatial frequencies indicate the frequencies characteristic of intensity variations in the image. For example, bright fibrillar structures spaced at distances of approximately 5 μm from each other would be represented by a spatial frequency of $\frac{1}{5\ \mu m} = 0.2\ \mu m^{-1}$, that is, one structure per 5 μm. While the power spectrum is typically presented as a function of spatial frequency, the spatial frequencies can be easily converted to wavelengths since wavelength is simply the reciprocal of spatial frequency. The wavelength representative of the structures of the above example would then be $\frac{1}{0.2\ \mu m^{-1}} = 5\ \mu m$. Except for the power spectrum plot, CytoSpectre uses wavelengths instead of spatial frequencies because they are often easier to interpret.

Inspecting the power spectrum can be useful for a number of reasons. By looking at the power spectrum plot, bright signals constrained to a particular spatial frequency (or wavelength) range may be spotted. These signals might represent particular structures of interest present in the

image, which could be extracted as the detail component. Even if they can often be detected using the automatic detail component detection settings (see Section 5.2.), specifying an expected range of wavelengths using the semi-automatic setting can allow detection of weaker signals. For example, one might observe that the spectrum features prominent signals between spatial frequencies of 1 μm^{-1} and 0.5 μm^{-1}. This corresponds to a wavelength range of 1 µm – 2 µm, which could now be used as the expected wavelength range for the semi-automatic detail component detection. By looking at the spectrum, the user might also notice that frequencies above (or below) a certain value do not contain any patterns but only random noise. Such random noise is always generated at the sensor and other electronics of the camera and the microscope, among other noise sources. Using the wavelength corresponding to this frequency as the minimum (or maximum) wavelength (see Section 5.2.) can then exclude the contributions of the random noise from the final analysis results.

8 Exporting results

8.1 Exporting to spreadsheets or text

To export morphological data (if any), orientation and wavelength statistics and used settings to an .xls spreadsheet or text file, first click **Export results** at the lower right corner of the CytoSpectre GUI. A dialog will pop-up. In the upper box, select **All analyzed images** to export data of all analyzed images or select **This image only** to export data of the selected image only. In the lower box, select **Spreadsheet summary** to write to a .xls file or select **Text summary** to write to a tab separated .txt file. Click **Export** and input a filename for the results file. Click **OK** and wait for the exporting to finish.

The resulting spreadsheet file includes the orientation and wavelength statistics for the mixed and detail components as well as morphological data (in case segmentation is enabled) on the first worksheet. These results are listed for each image and each cell (in case segmentation is enabled). The second worksheet contains the settings used for each image. The text file has all the same data as the .xls file but the analysis settings for each cell and image are appended to the end of each row, as .txt files do not have multiple worksheets. The text file is in tab separated format. See Section 7 for a description of all the statistics computed by CytoSpectre.

8.2 Exporting raw data

To export raw orientation and wavelength distributions, first click **Export results** at the lower right corner of the CytoSpectre GUI. A dialog will pop-up. In the upper box, select **All analyzed images** to export data of all analyzed images or select **This image only** to export data of the selected image only. In the lower box, select **Raw distributions**. Click **Export** and input a filename for the data file. Click **OK** and wait for the exporting to finish.

The resulting output file contains the orientation distribution values and the corresponding indices (i.e. angles in radians) for the mixed and detail components. These data are followed by the wavelength distribution values and corresponding indices (i.e. wavelengths given according to the selected length unit) for the mixed and detail component. Each row of values or indices is

preceded by a header line, which contains the image name, cell index and a string specifying which values or indices are listed on the following row. The file thus has the following 16 lines in tab separated format per image or cell:

<imagename>   <cell index>   Mixed_orientation_values
<values of the mixed component orientation distribution>
<imagename>   <cell index>   Mixed_orientation_index (radian)
<indices in radians for the mixed component orientation distribution>
<imagename>   <cell index>   Detail_orientation_values
<values of the detail component orientation distribution>
<imagename>   <cell index>   Detail_orientation_index (radian)
<indices in radians for the detail component orientation distribution>
<imagename>   <cell index>   Mixed_wavelength_values
<values of the mixed component wavelength distribution>
<imagename>   <cell index>   Mixed_wavelength_index (<length unit>)
<indices in given length unit for the mixed component wavelength distribution>
<imagename>   <cell index>   Detail_wavelength_values
<values of the detail component wavelength distribution>
<imagename>   <cell index>   Detail_wavelength_index (<length unit>)
<indices in given length unit for the detail component wavelength distribution>

Note: *Raw data files can be very large (typically tens of megabytes per image, depending on settings and image size). They are mainly intended to be used by computational experts wishing to utilize the data produced by CytoSpectre for some customized application.*

8.3 Exporting plots as images

To export the orientation and wavelength distribution plots as images, first click **Export results** at the lower right corner of the CytoSpectre GUI. A dialog will pop up. In the upper box, select **All analyzed images** to export data of all analyzed images or select **This image only** to export data of the selected image only. In the lower box, select **Figures**. Click **Export** and input a filename prefix for the files. Click **OK** and wait for the exporting to finish.

The names of the four resulting files have the following formats:
<prefix>_<imagename>_<cell index>_orientation_mixed.tif
<prefix>_<imagename>_<cell index>_wavelength_mixed.tif
<prefix>_<imagename>_<cell index>_orientation_detail.tif
<prefix>_<imagename>_<cell index>_wavelength_detail.tif

Each file shows the corresponding distribution plot for cell numbered <cell index> in image <imagename>.

9 Operation via the MATLAB command line

In addition to standalone operation using the deployed Windows application, CytoSpectre can be run via MATLAB. The graphical user interface can be started by running **CytoSpectreGUI.m**. Functionality of the software is then identical to the standalone deployed application (see Sections 5-8).

It is also possible to run the spectral analysis programmatically via the MATLAB command line without using the user interface. This can be useful, if you wish to run CytoSpectre on a server or integrate the analysis into a customized analysis pipeline or external software. The main analysis can be run for a single image using the function **CytoSpectre** with the following arguments:

`result = CytoSpectre(inputimage,settings,segmentationmask)`

`inputimage` is a numerical matrix containing the image to analyze.

`settings` is an optional analysis settings structure. If this argument is provided, the specified settings are used instead of default values. Otherwise the analysis is run using default settings (see Section 5.2. for the default value of each setting). The structure can contain one or more of the following fields, each controlling the corresponding setting listed in Section 5.2.:

`magnification`	Magnification.
`camerapixelsize`	Camera pixel size. Should be given in the units preferred for the results and wavelength settings as well. For example, if you want the final results in millimeters and the pixel size of your camera is 6 µm, set this value to 0.006.
`targetchannel`	Target channel (1=red, 2=green, 3=blue, 4=combined).
`rotatemixed`	Rotate mixed component orientation by 90 degrees (1=yes, 0=no).
`rotatedetail`	Rotate detail component orientation by 90 degrees (1=yes, 0=no).
`resolutionparameter`	Spectral resolution/noise (1=minimum noise, 2=low noise, 3=balanced, 4=high resolution, 5=maximum resolution).
`lowlimit`	Minimum wavelength.
`highlimit`	Maximum wavelength.
`excludelow`	Exclude wavelength band, lower limit.
`excludehigh`	Exclude wavelength band, higher limit.
`lowguess`	Detail component wavelength band, lower limit.
`highguess`	Detail component wavelength band, higher limit.
`segmentlength`	Segment length.
`alpha`	Significance level.
`angleslack`	Angular tolerance.
`convergence_threshold`	Convergence limit.
`maxiterations`	Max. iterations.
`sigma_prior_angle`	Prior width, angle.

`segmentationmask` is an optional mask image matrix with logical true for pixels belonging to the target cell and logical false for pixels belonging to background. Size of the image should be

the same as the size of `inputimage`. If this argument is provided, segmentation is enabled. Otherwise segmentation is disabled.

`results` is a structure containing the results of the analysis (see Sections 7.3.-7.5. for details on the estimated parameters). The structure contains the following fields:

`orientation_mixed`	Orientation distribution and summary statistics for mixed component.
`orientation_detail`	Orientation distribution and summary statistics for detail component.
`distance_mixed`	Wavelength distribution and summary statistics for mixed component.
`distance_detail`	Wavelength distribution and summary statistics for detail component.
`detailquality`	Detail component signal-to-noise ratio in dB.

Each field (except for `detailquality`) in turn contains a structure with the distribution values, corresponding angle or wavelength index and the estimated summary statistic values.

10 Troubleshooting

**Problem**	**Possible solutions**
Disk space runs out during analysis.	CytoSpectre creates temporary files to reduce the amount of required RAM. Depending on the settings, these can require even more disk space than the original images. If your images are located e.g. on a memory stick with limited free space, CytoSpectre can run out of disk space for the temporary files. Go to **Preferences -> General settings** and change the **Location of temporary files** to a directory that has plenty of free disk space.
The analysis takes a long time to run.	Computation time increases with increasing image size. Use of binning (i.e. averaging adjacent pixels) while acquiring the images can decrease image size, often without significant loss of resolution. Image processing software can also be used after image acquisition to resize images. Binning or image resizing is only encouraged if sufficient resolution is still retained.<br><br>Faster hardware can decrease computation times. We recommend at least an Intel CORE™ i5 processor or similar.<br><br>The **Spectral resolution/noise** setting can be modified to decrease computation time. The

	maximum or high resolution settings allow faster computations than the low or minimum noise settings.<br><br>The **Max. iterations** setting can be modified to decrease computation time. Setting a lower value, e.g. 5 can make computations significantly faster but it may also decrease detail component detection performance. If your image has no interesting details and you are only analyzing the mixed component, the value can be safely set to 1.
Wavelength statistics differ from expected values.	Make sure the **Magnification** and **Camera pixel size** settings are correct.<br><br>Magnification should equal the combined magnification of the objective and the camera adapter or any other optical components that affect the overall magnification. For example, if your images have been obtained using an objective with 40X magnification and a microscope equipped with a 0.63X camera adapter, the combined magnification is 40 x 0.63 = 25.2. For a 1X camera adapter, the magnification using the same objective would simply be 40X.<br><br>Camera pixel size should be the effective pixels size of the camera, taking into account binning, if enabled. Binning means averaging adjacent pixels into one and is typically expressed as 1x1, 2x2 etc. in image acquisition software. One way to find out if binning has been used is to compare the amount of pixels in the images and the maximum image size given in the technical specifications of the camera. For example, if the maximum image size is 2584x1936 but the size of your images is 1292x968, 2x2 binning has been probably used during image acquisition.
The orientation distribution features a sharp vertical or horizontal peak.	Sometimes scale bars can cause powerful artefact signals, typically manifested as either horizontal or vertical peaks in the orientation distribution. For this reason, we recommend analyzing images without scale bars. In some cases, this effect can be partly remedied by

	increasing the **minimum wavelength** value.<br><br>Some cameras may also produce vertical or horizontal artefact lines, which are then reflected as peaks in the orientation distribution. Such artefacts can typically be seen as bright spots at the high-frequency end of the power spectrum. As in the case of scale bars, effects caused by such artefacts can sometimes be removed by increasing the **minimum wavelength** value.
Exporting results to a spreadsheet fails.	There might be a compatibility issue between CytoSpectre and your Microsoft Excel installation. One way to circumvent this issue is to export the results into a plain text file, which can then be easily imported to Microsoft Excel or another program.

11 License agreements

11.1 MATLAB source code form

By using CytoSpectre in MATLAB source code form, you agree to the terms of the disclaimer below, the GNU General Public License (GPL) and The Circular Statistics Toolbox for MATLAB license. All original CytoSpectre .m-files are licensed under the GPL license, see the file GPL_license.txt for details. All .m-files belonging to The Circular Statistics Toolbox for MATLAB are licensed under the corresponding license, see the file CircStats_license.txt for details.

THIS SOFTWARE IS PROVIDED BY THE COPYRIGHT HOLDERS AND CONTRIBUTORS "AS IS" AND ANY EXPRESS OR IMPLIED WARRANTIES, INCLUDING, BUT NOT LIMITED TO, THE IMPLIED WARRANTIES OF MERCHANTABILITY AND FITNESS FOR A PARTICULAR PURPOSE ARE DISCLAIMED. IN NO EVENT SHALL THE COPYRIGHT OWNER OR CONTRIBUTORS BE LIABLE FOR ANY DIRECT, INDIRECT, INCIDENTAL, SPECIAL, EXEMPLARY, OR CONSEQUENTIAL DAMAGES (INCLUDING, BUT NOT LIMITED TO, PROCUREMENT OF SUBSTITUTE GOODS OR SERVICES; LOSS OF USE, DATA, OR PROFITS; OR BUSINESS INTERRUPTION) HOWEVER CAUSED AND ON ANY THEORY OF LIABILITY, WHETHER IN CONTRACT, STRICT LIABILITY, OR TORT (INCLUDING NEGLIGENCE OR OTHERWISE) ARISING IN ANY WAY OUT OF THE USE OF THIS SOFTWARE, EVEN IF ADVISED OF THE POSSIBILITY OF SUCH DAMAGE. MATHWORKS AND ITS LICENSORS ARE EXCLUDED FROM ALL LIABILITY FOR DAMAGES OR ANY OBLIGATION TO PROVIDE REMEDIAL ACTIONS.

11.2 Deployed application

By using the CytoSpectre deployed application, you agree to the terms of the disclaimer below, the Matlab Compiler Runtime (MCR) libraries license, applicable parts of The MathWorks, Inc. Software License Agreement and the Circular Statistics Toolbox for Matlab license. See the files MCR_license.txt, MATLAB_license.txt and CircStats_license.txt, provided with the installation package, for details. Using the CytoSpectre deployed application for commercial purposes is prohibited. See The MathWorks, Inc. Software License Agreement - Academic Installation and Use Addendum for details.

THIS SOFTWARE IS PROVIDED BY THE COPYRIGHT HOLDERS AND CONTRIBUTORS "AS IS" AND ANY EXPRESS OR IMPLIED WARRANTIES, INCLUDING, BUT NOT LIMITED TO, THE IMPLIED WARRANTIES OF MERCHANTABILITY AND FITNESS FOR A PARTICULAR PURPOSE ARE DISCLAIMED. IN NO EVENT SHALL THE COPYRIGHT OWNER OR CONTRIBUTORS BE LIABLE FOR ANY DIRECT, INDIRECT, INCIDENTAL, SPECIAL, EXEMPLARY, OR CONSEQUENTIAL DAMAGES (INCLUDING, BUT NOT LIMITED TO, PROCUREMENT OF SUBSTITUTE GOODS OR SERVICES; LOSS OF USE, DATA, OR PROFITS; OR BUSINESS INTERRUPTION) HOWEVER CAUSED AND ON ANY THEORY OF LIABILITY, WHETHER IN CONTRACT, STRICT LIABILITY, OR TORT (INCLUDING NEGLIGENCE OR OTHERWISE) ARISING IN ANY WAY OUT OF THE USE OF THIS SOFTWARE, EVEN IF ADVISED OF THE POSSIBILITY OF SUCH DAMAGE. MATHWORKS AND ITS LICENSORS ARE EXCLUDED FROM ALL LIABILITY FOR DAMAGES OR ANY OBLIGATION TO PROVIDE REMEDIAL ACTIONS.
