# Supplementary material for: CytoSpectre: a tool for spectral analysis of oriented structures on cellular and subcellular levels
Source: BMC Bioinformatics. 2015 Oct 26;16:344. doi: 10.1186/s12859-015-0782-y (PMC4624586; doi:10.1186/s12859-015-0782-y)
Supplement: Additional file 4: — Supplementary information. This file contains detailed descriptions of computational and experimental methods as well as results and discussion of the sensitivity analysis. (PDF 715 kb) [file 12859_2015_782_MOESM4_ESM.pdf]

Supplementary information

CytoSpectre: a tool for spectral analysis of oriented structures on cellular and subcellular levels

Kimmo Kartasalo, Risto-Pekka Pölönen, Marisa Ojala, Jyrki Rasku, Jukka Lekkala, Katriina Aalto-Setälä, Pasi Kallio

Supplementary results

Sensitivity analysis of adjustable parameters

In order to test how sensitive CytoSpectre is to the exact values of user-adjustable parameters, we re-analyzed the simulated fibril dataset using different combinations of adjustable parameters. The value of a single parameter was varied at a time within reasonable limits while keeping all other settings at their default values. The value of the maximum iterations parameter N_{max} was not varied, as the number of iterations performed during detail component extraction is mainly controlled by the convergence threshold parameter, as long as the maximum iterations parameter has been set at a high enough value. The parameters and their tested values are given in Table S1 with the default values made bold. The values estimated for the detail component were then compared with the true values. Relative change in RMS error was used to quantify the impact of different parameter values, allowing comparison between the different estimated parameters. The relative change was expressed as a percentage of the RMS error obtained using default settings. The results are visualized for each parameter in Figures S1-S6. Full numerical data for the experiment are provided in Additional file 9.

Bases on the sensitivity analysis, most of the adjustable parameters may be fairly safely assumed to have relatively little impact on the results produced by CytoSpectre, as long as the adjustable values are kept within certain limits. The apparently large variations in RMS error observed for the mean orientation in the case of several parameters are mainly explained by the low absolute errors of this parameter. It is doubtful that an increase in the mean orientation error from e.g. two degrees to three degrees would be catastrophic in typical applications, even though the relative change would be 50 %. We expect that in most cases, errors of this magnitude are overshadowed by biological or technical variation. For most of the adjustable parameters and their tested values, the increases in RMS error were less than 5 % and exceeded 10 % only near the limits of the tested parameter value ranges. The prior size parameter *b* is the only exception, as increasing its value beyond 0.6 rapidly increased the RMS errors of most of the estimated parameters. However, there was still a relatively stable range of values for this parameter between 0.2 and 0.6, where the increase in RMS error stays below 10 % for all estimated parameters. Based on these results, we

conclude that tuning of the adjustable parameters by the user is rarely necessary and the default values can be expected to represent an acceptable compromise for most images.

Table S1: Adjustable parameters and their tested values, with the default values made bold.

Parameter	Description	Values tested
Resolution parameter *W*	Controls the tradeoff between spectral resolution and noise during spectral estimation.	1 (Minimum noise), 2 (Low noise), **3 (Balanced)**, 4 (High resolution), 5 (Maximum resolution)
Segment length *L*	Controls the length of the spectral segment used during estimation of spectral background and detail component main orientation.	0.02, 0.04, 0.06, 0.08, **0.10**, 0.12, 0.14, 0.16, 0.18, 0.20
Significance level α	Significance level used for statistical testing during estimation of detail component main orientation.	0.01, 0.02, 0.03, 0.04, **0.05**, 0.06, 0.07, 0.08, 0.09, 0.10
Angular tolerance ε	Controls the number of angular spectral bins used during estimation of detail component wavelength range.	0, 5, **10**, 15, 20, 25, 30, 35, 40, 45
Convergence threshold τ	Controls the stopping threshold for the iterative detail component extraction procedure.	**1**, 2, 3, 4, 5, 6, 7, 8, 9, 10
Prior size *b*	Controls the initial angular width of the Gaussian selector function used during the iterative detail component extraction procedure.	0.1, 0.2, 0.3, 0.4, **0.5**, 0.6, 0.7, 0.8, 0.9, 1.0

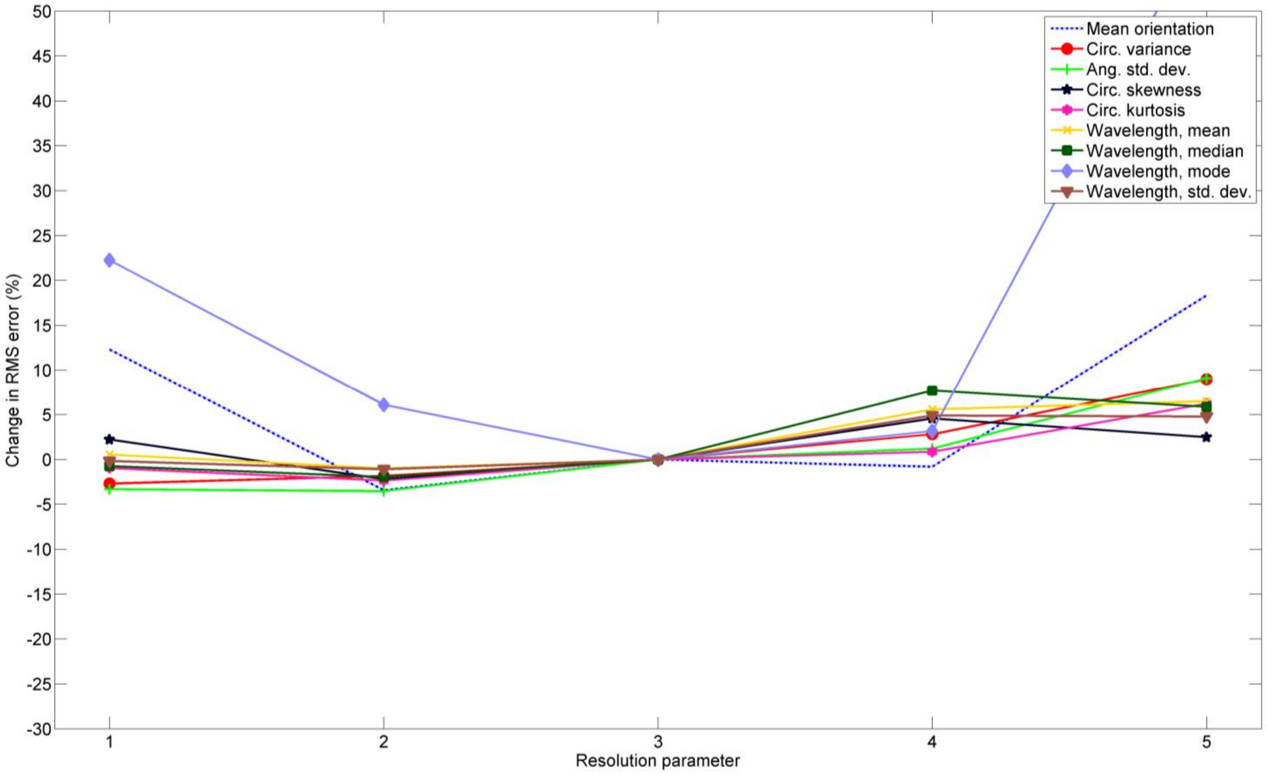

Figure S1: Change in the RMS error of estimated parameters for different resolution parameter *W* values relative to the default setting of 3 (Balanced). Values exceeding 50 % are excluded.

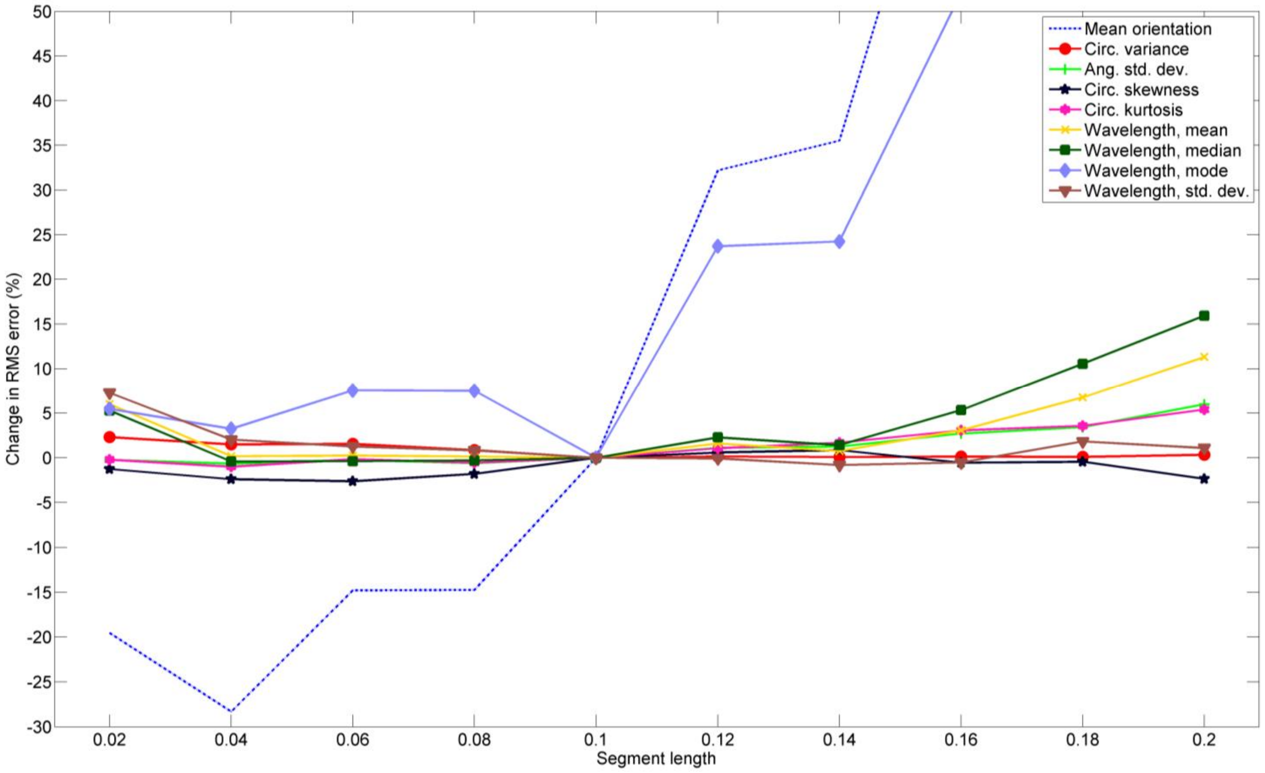

Figure S2: Change in the RMS error of estimated parameters for different segment length *L* values relative to the default setting of 0.10. Values exceeding 50 % are excluded.

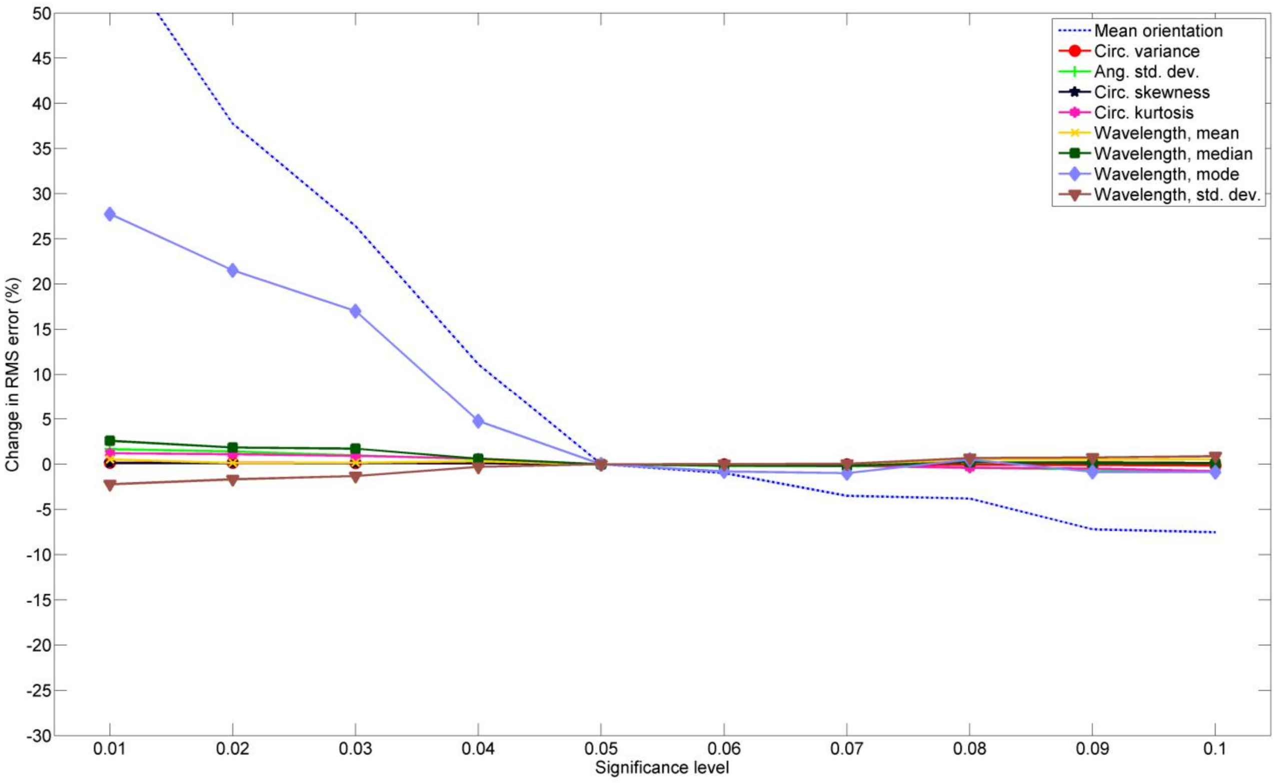

Figure S3: Change in the RMS error of estimated parameters for different significance level α values relative to the default setting of 0.05. Values exceeding 50 % are excluded.

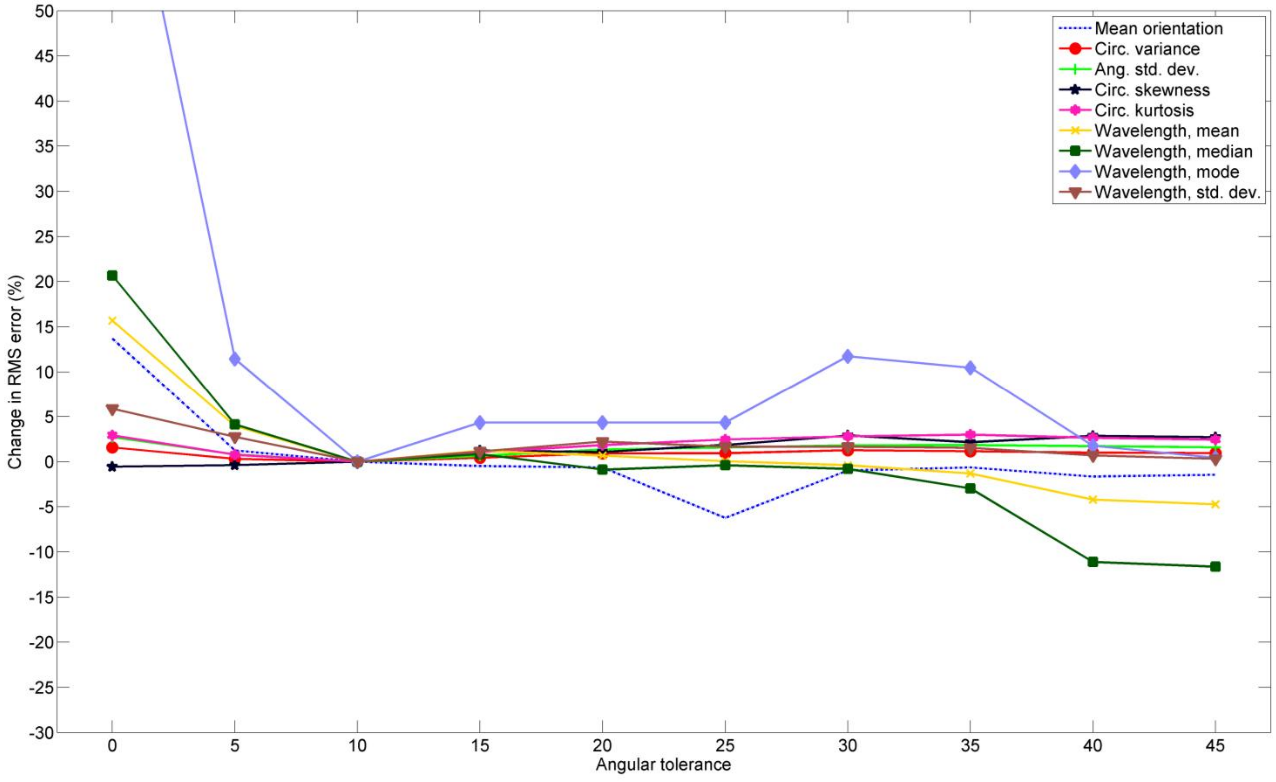

Figure S4: Change in the RMS error of estimated parameters for different angular tolerance ε values relative to the default setting of 10 degrees. Values exceeding 50 % are excluded.

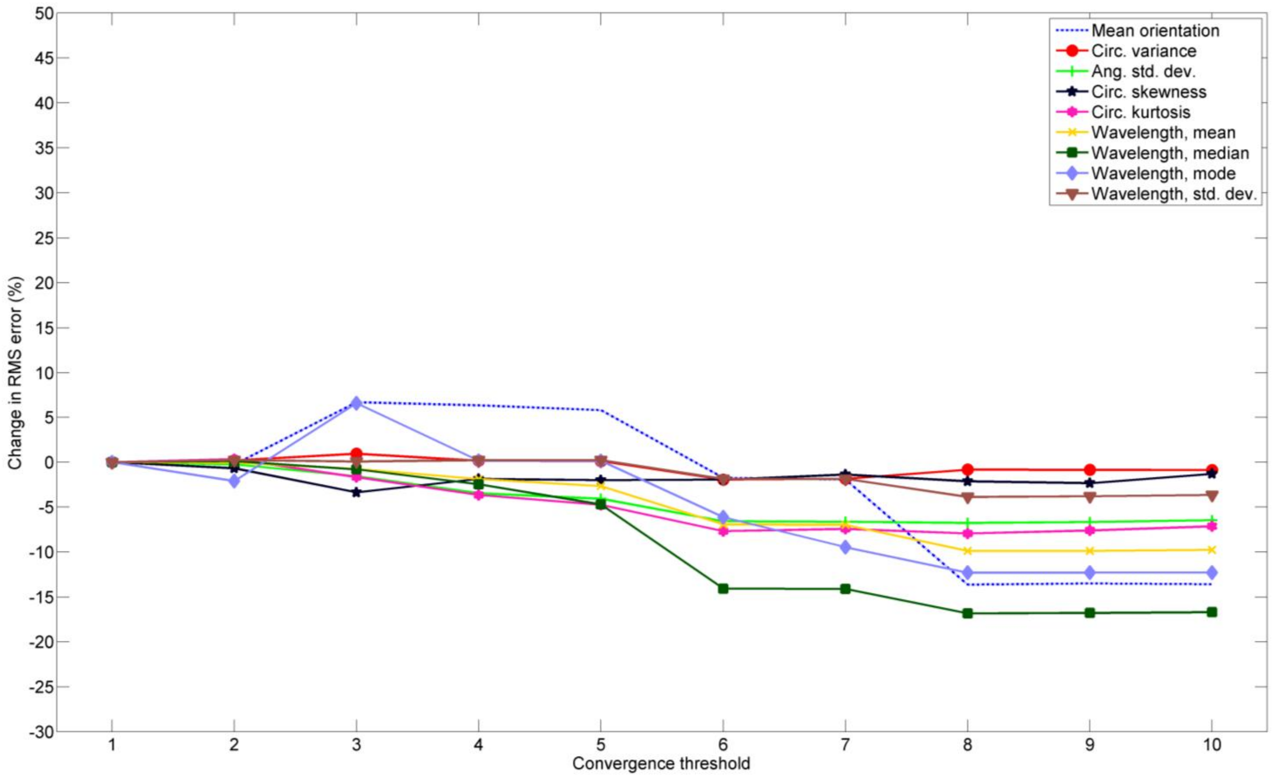

Figure S5: Change in the RMS error of estimated parameters for convergence threshold τ values relative to the default setting of 1 degree. Values exceeding 50 % are excluded.

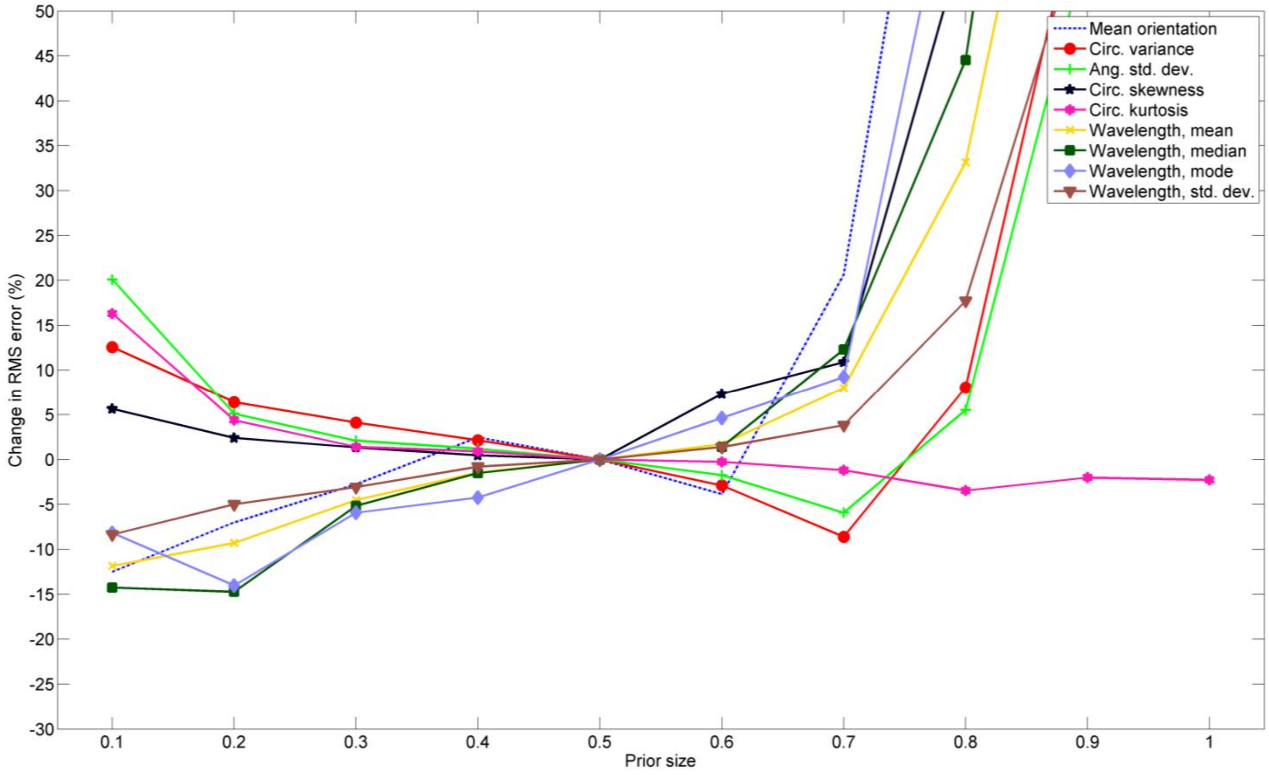

Figure S6: Change in the RMS error of estimated parameters for different prior size *b* values relative to the default setting of 0.50. Values exceeding 50 % are excluded.

Supplementary computational methods

Calculation of morphological parameters

If cell segmentation is enabled, a number of parameters summarizing the morphological features of each cell are calculated using the *regionprops* function of MATLAB. If segmentation is disabled, this step of the analysis is skipped. The parameters include:

Center X: The x-coordinate of the cell's centroid.

Center Y: The y-coordinate of the cell's centroid.

Area: The physical area covered by the cell.

Perimeter: The physical perimeter of the cell i.e. the distance along the outline of the cell.

Major axis: The length of the major axis of an ellipse that has the same normalized second central moments as the cell region i.e. the 'length' of the cell along its longitudinal axis.

Minor axis: The length of the minor axis of an ellipse that has the same normalized second central moments as the cell region i.e. the 'width' of the cell along its transverse axis.

Eccentricity: The ratio of the distance between the foci of an ellipse that has the same normalized second central moments as the cell region and the major axis length of the ellipse. Eccentricity values lie in the interval [0, 1] with a value of zero corresponding to a circle and a value of one corresponding to a line segment. In other words, cells that have an elongated shape have a higher eccentricity than cells whose shape is round.

Solidity: The ratio of cell area and the area of the cell's convex hull. Solidity values lie in the interval (0, 1] with smoothly shaped cells having a high value and cells with protrusions having a low value.

Centroid coordinates, area, perimeter, major axis length and minor axis length are converted from pixels to physical units in accordance with the size of a single image pixel.

Spectral estimation

Spectral estimation is performed with Welch's method, also known as Weighted Overlapped Segment Averaging (WOSA) [20]. If cell segmentation is enabled, background pixels are set to zero prior to spectral estimation. Otherwise, the whole image is analyzed. Mean is first subtracted from the image and the image is then divided into N_S square segments with approximately 50 % overlap between adjacent segments. The size of the segments, controlling the tradeoff between spectral resolution and stability, can be selected by the user by adjusting the spectral resolution parameter W. The options include ⅕, ¼, ⅓, ½ or 1/1 of the size of the shorter image dimension. The ⅕ option produces smooth spectra featuring small amounts of random fluctuations at the expense of decreased spectral resolution while the 1/1 option produces noisy spectral estimates with maximal spectral resolution. The other options represent different compromises between the

two extremes. In most cases, a balanced setting such as ⅓ is appropriate. Each segment is multiplied by a circularly symmetric Hann window to reduce spectral leakage and the discrete Fourier transform (DFT) of the segments is then computed with the Fast Fourier Transform (FFT) algorithm. Before computing the DFT, the segment is zero-padded either to a size equaling the next power of two larger than the original segment size or to a minimum size of 1024 x 1024, whichever is larger. The power spectral density (PSD) of each segment is obtained as the squared modulus of the corresponding DFT and the final spectral estimate is formed as the arithmetic mean of the power spectra of all segments. The conventional shifting operation is then performed to place the lowest frequencies at the center of the spectrum. Finally, the resulting power spectral estimate is normalized such that its sum is forced to correspond to the variance of the input image, in accordance with Parseval's theorem.

The power spectrum is transformed into polar coordinates for easier processing during later analysis steps. The original 2D power spectrum *F(u,v),* expressed in Cartesian coordinates, can be transformed into *P(f,*θ), expressed in polar coordinates, according to the polar coordinate transform:

$$\theta = \tan^{-1}\frac{v}{u} \tag{1}$$

$$f = \sqrt{u^2 + v^2} \tag{2}$$

The transform is performed using bilinear interpolation. The number of spectral elements in the interpolated polar power spectrum is selected so as to retain or exceed the resolution of the Cartesian spectrum along both dimensions at all spatial frequencies below the Nyquist frequency $f_{Nyquist}$. The numbers of bins required along the two dimensions are calculated based on the number of spatial frequency bins N_{uv} in the Cartesian representation. Neither the zero spatial frequency bin nor frequencies above $f_{Nyquist}$ are included in the power spectrum after the polar coordinate transform. Moreover, only angles from 0 to 180 degrees (i.e. the upper half circle in the Cartesian power spectrum) are retained, since the lower and upper half circles of the Cartesian power spectrum are symmetric. For the angular dimension, the required number of angle bins in the polar power spectrum is calculated based on the circumference of a circle whose radius corresponds to the number of spatial frequency bins between zero frequency and $f_{Nyquist}$ as:

$$N_\theta = \left\lceil \pi \frac{N_{uv}}{2} \right\rceil \tag{3}$$

For the spatial frequency dimension, the required number of frequency bins in the polar power spectrum is:

$$N_f = \left\lfloor \frac{N_{uv}}{2} \right\rfloor \tag{4}$$

Since the density of spectral elements in the Cartesian power spectrum is different at different spatial frequencies i.e. at different distances from the origin, the values of the polar power spectrum need to be adjusted by a scaling factor that is dependent on spatial frequency. An adjusted element of the polar power spectrum $P(f,\theta)$ is obtained as:

$$\overline{P}(f, \theta) = \frac{\pi}{2} \frac{f}{f_{Nyquist}} P(f, \theta) \tag{5}$$

Estimation of spectral background

An estimate of spectral background is formed based on the polar power spectrum estimate to allow detection of spectral signals based on their intensity relative to the background. The background spectrum estimate represents image features, such as noise, which are not of interest when analyzing the structures present in the image. Spectral signals representing meaningful details and structures of interest in the image appear as peaks and local fluctuations on top of the smoother background spectrum. It has been widely reported, that the rotationally averaged power spectra of natural images tend to fall with increasing spatial frequency f according to a power law $1/f^{\alpha}$ with varying values of α observed in different situations [41][42][43][44][45]. That is, the low spatial frequency region of the power spectrum tends to dominate the high spatial frequency region. An estimate of the general shape of the spectral background is needed in order to differentiate signals from the background.

The background spectrum is estimated based on the polar power spectrum using averaging over all orientations and a smoothing procedure. The advantage of not using a parametric model for the background spectrum is greater flexibility in terms of applications and imaging systems. The downside is that since the background spectrum estimate is based on an estimate of the power spectrum of the actual image being analyzed, it is biased by spectral peaks representing signals of interest. The bias is reduced by smoothing to remove most of the spectral peaks and to retain only the general shape of the spectral background. First, the polar power spectrum is averaged over all orientations to obtain a one-dimensional spectrum. The one-dimensional spectrum is then transformed to log-log scale and linear interpolation, retaining the original number of data points, is performed to obtain equidistant data points along the spatial frequency axis. Smoothing via robust

local regression using weighted linear least squares and a first degree polynomial model (robust lowess) is then performed. The smoothing procedure is implemented using MATLAB's *smooth* function. The method decreases the effect of outliers (i.e. elements probably belonging to spectral peaks originating from genuine signals) by assigning zero weight to data outside six mean absolute deviations. By default, the span parameter *L* associated with the smoothing method is 10 % of the length of the one-dimensional spectrum (i.e. the number of spatial frequency bins in the polar power spectrum). The value of the span parameter can also be adjusted by the user, but the analysis is not sensitive to the exact value. Smoothing is performed in log-log space to effectively vary the span depending on spatial frequency, allowing high frequency signal peaks to be suppressed while retaining the general spectral shape, especially the typically narrow high intensity peak near zero frequency, at low frequencies. This effect is obtained because the log-log transform followed by linear interpolation effectively corresponds to upsampling at low frequencies and downsampling at high frequencies. Finally, the smoothed one-dimensional spectrum is converted back to linear scale and tiled to form a two-dimensional polar background spectrum whose size is identical to the original polar power spectrum. The background spectrum formed this way is thus isotropic and does not introduce any orientational bias during later analysis steps.

Probabilistic representation of the power spectrum

Based on the estimated background spectrum, the values of the polar power spectrum are converted to probabilities. After the conversion, each element $\overline{P}(f,\theta)$ of the spectrum is assigned a value representing the probability that the element belongs to a signal. In this context, 'a signal' should be understood loosely as some feature of the image represented by power spectral values larger than the corresponding values of the estimated spectral background. That is, elements of the power spectrum having a high magnitude relative to the corresponding element of the background spectrum should be assigned a high probability value, while spectral elements with values close to the background should be assigned low probabilities. To convert the values of the polar power spectrum to probabilities, we first utilize a normalized WOSA spectrum, defined as [46]:

$$R(f,\theta) = \frac{\overline{P}(f,\theta)}{S(f,\theta)} \tag{6}$$

where $S(f,\theta)$ is the background spectrum estimate. In the assumption of vanishing correlations caused by the windowing and overlapping process, the normalized WOSA spectrum is gamma distributed with σ = 1/N_S and h = N_S [46][47]. The probability $Z(f,\theta)$ that the element $\overline{P}(f,\theta)$ represents a signal is then obtained as the value of the gamma cumulative density function evaluated at the corresponding value of $R(f,\theta)$:

$$Z(f,\theta) = \frac{1}{\sigma^h \Gamma(h)} \int_0^{R(f,\theta)} t^{h-1} e^{\frac{-t}{\sigma}} dt \quad (7)$$

where Γ(·) is the Gamma function.

Selection of spatial frequency cutoffs

Some of the lowest spatial frequencies are usually excluded from analysis because the definition of angle is very coarse near the zero spatial frequency i.e. close to the origin of the Cartesian power spectrum [5] and the information present at the lowest frequencies is typically not of interest [6]. Even though the number of angle bins in the polar power spectrum does not depend on spatial frequency, the values of the spectral elements close to zero spatial frequency have nevertheless been interpolated from a very small number of original data points in Cartesian space. Another reason for excluding some of the lowest spatial frequency bins are artefacts caused by the windowing process [21]. The exact cutoff frequency has typically been selected based on experimentation with different values [5][6][7][9][21]. In CytoSpectre, the lowest spatial frequencies are by default excluded based on a fixed minimum angular resolution requirement of five degrees. Based on this criterion and the Cartesian representation of the power spectrum, a suitable lower spatial frequency cutoff f_L can be selected by excluding a circular element of the power spectrum, centered on the zero frequency bin. By requiring the number of bins along the circumference of this circle to be such that the angular resolution criterion is fulfilled, the corresponding number of spatial frequency bins to exclude, starting from the origin or zero frequency, can be obtained as:

$$N_L = \left\lceil \frac{36}{\pi} \right\rceil = 12 \quad (8)$$

The actual spatial frequency value corresponding to the 12th bin then depends on the physical dimensions of the image. Provided that random noise is not an issue, specifying a value for the upper cutoff frequency f_H is somewhat arbitrary [9]. By default, the highest spatial frequency included in the analysis in CytoSpectre is limited only by the Nyquist frequency. Both of the cutoff frequencies can also be freely modified by the user by selecting corresponding cutoff wavelengths of choice. This may be desirable e.g. to exclude high-frequency noise or to focus the analysis on a particular wavelength range of interest.

Mixed component extraction

The mixed component represents all features of the image present in the range of spatial frequencies dictated by the upper and lower cutoff frequencies. To extract the parts of the

spectrum associated with the mixed component, all spectral elements outside the cutoff frequencies are set to zero. The mixed component can thus be expressed as:

$$P_M(f,\theta) = \begin{cases} \overline{P}(f,\theta), & f_L \leq f \leq f_H \\ 0, & \text{otherwise} \end{cases} \tag{9}$$

Estimation of initial parameter values for detail component detection

The detail component represents some particular group of structures with a limited range of spatial frequencies and/or orientations, such as intracellular fibrils of given size. Such structures are reflected in the power spectrum as more or less contiguous regions of high intensity relative to background. The aim of the detail component extraction procedure is to separate these regions from the power spectrum. CytoSpectre uses an iterative method, bearing some resemblance to a Bayesian classifier and segmentation algorithms based on region growing, to separate the spectral region corresponding to the detail component. Before the iterative procedure, a number of initial parameter values are estimated.

First, the dominant orientation of the low frequency range of the power spectrum is estimated using a sliding window approach. Mean orientation is first computed for each spatial frequency bin of the polar power spectrum using the corresponding function of the CircStat toolbox [22], excluding bins below and above the lower and higher cutoff frequencies, respectively. Angle doubling is performed to account for the axial nature of the data [22] and the mean orientation angle is afterwards divided by two. The mean orientation values are then transformed to absolute differences relative to the mean orientation of the first bin to avoid problems near zero or 180 degrees. A sliding window, whose length is equal to 10 % of the total number of spatial frequency bins by default, is then applied to detect the spatial frequency at which a transition in the mean direction takes place. The window is incrementally moved by one bin at a time, starting from the low frequency end, towards higher frequencies, and a Mann-Whitney test is performed at each location to test if the mean orientations within the bins overlapping the window differ significantly from all previous bins. By default, a significance level of 0.05 is used to detect the transition. The window length L and the significance level α can also be adjusted by the user, but the analysis is not sensitive to the exact values of these parameters. Mean orientation $\bar{\theta}$ within the range of spatial frequencies defined by the lower cutoff frequency and the detected transition frequency is then calculated as described above. If no transition is detected, the whole spatial frequency range between the cutoff frequencies is used for calculating the mean direction. The obtained mean direction represents the dominant orientation at low frequencies, which is mostly dominated by

gross properties of the image, such as the overall orientation of a complete cell imaged at high magnification.

The spatial frequency range of the detail component is estimated by first obtaining a slice of the polar power spectrum along the expected mean orientation of the detail component, followed by a peak fitting procedure. By default, the slice contains the elements of the polar power spectrum whose orientations are within ten degrees from the dominant orientation estimated during the previous step. This is the case for typical structures, which exhibit intensity variations along orientations that are transverse to the longitudinal axis of the structures. Because orientations in the power spectrum reflect the orientation of intensity variation in the image, spectral signals originating from such structures are offset by 90 degrees relative to the orientation of the structures themselves. By default, this offset is corrected during the analysis. For structures exhibiting intensity variations along their longitudinal axis, such as myofibrils and their characteristic striated intensity patterns, the correction should not be made, as the orientations seen in the power spectrum directly correspond to the orientation of the structures of interest. The selection between these two options can be accomplished by adjusting the analysis settings accordingly. In the latter case, the spectral slice is obtained along orientations within ten degrees from the orientation transverse to the dominant low frequency orientation. The default tolerance of ten degrees can also be adjusted by the user by modifying the value of the angular tolerance parameter ε, but the exact value is not critical for the analysis. Averaging over the selected orientations is then performed to obtain a one-dimensional vector of spectral values.

Next, the values of corresponding elements of the background spectrum are subtracted from the one-dimensional spectral slice. A Gaussian peak is then fitted to this background subtracted spectrum using a non-linear least squares fitting routine. If the semi-automatic detail component detection setting is enabled and the user supplies a guess for the wavelength range of the detail component, spatial frequencies corresponding to the user-specified limits are used to constrain the peak fitting procedure. In this case, the mean of the Gaussian peak is constrained between the user-specified limits and the standard deviation of the Gaussian is constrained between ¼ and ½ of the spatial frequency range defined by the limits. Peak height is constrained between zero and the actual maximum value of the background subtracted spectrum between the user-specified limits. If the fully automatic setting is used and no guesses are available, the highest peak between lower and upper cutoff frequencies is searched. The peak is then treated as if it were a perfect Gaussian to find locations at distances corresponding to two standard deviations from the peak location. In other words, nearest locations at both sides of the peak with values less than e^2 times the peak height are searched. The spatial frequencies corresponding to these locations are then used to constrain the peak fitting procedure as in the case of user-specified limits. In both cases,

the initial values of all three parameters are set halfway between the lower and upper limits. The mean $\bar{f}$ and standard deviation σ_f produced by the fitting procedure, reflecting the estimated spatial frequency range of the detail component, are then stored.

After the expected mean orientation $\bar{\theta}$ and spatial frequency range of the detail component have been estimated, an initial estimate of the spread of the orientation distribution σ_θ is computed. This is accomplished by calculating the angular standard deviation of the polar power spectrum at spatial frequencies within two standard deviations from the mean of the Gaussian peak. The values obtained for $\bar{f}$ and σ_f during the previous peak fitting step are used here. The angular standard deviation is computed with the corresponding function from the CircStat toolbox [22].

Detail component extraction

The detail component extraction procedure is inspired by a Bayesian classifier. The previously formed probabilistic form of the polar power spectrum $Z(f,\theta)$ is multiplied by a two-dimensional truncated Gaussian selector function $K(f,\theta)$ and the resulting values $X(f,\theta)$ are interpreted as probabilities. Each value now represents the probability that the corresponding element of the power spectrum belongs to the detail component spectral region $P_D(f,\theta)$. This should be understood as a purely methodological concept, since the values do not necessarily represent *bona fide* probabilities. If expressed within the Bayesian classifier framework and terminology, the role of the probabilistic power spectrum corresponds to the data or observations while the Gaussian selector function is used similarly to a prior. In keeping with the Bayesian classifier interpretation, the values resulting from the multiplication step are treated as if they were posterior probabilities. Spectral elements with values exceeding 0.5 are therefore accepted as part of the detail component spectral region, as their 'probability' of belonging to the detail component is higher than the 'probability' of the complement case of not belonging to the detail component (i.e. probability of belonging to the background).

The values estimated during the previous analysis step are used to initialize the two-dimensional Gaussian selector function $K_0(f,\theta)$. The standard deviation of the Gaussian selector function along the spatial frequency axis is set to:

$$\sigma_{K,f} = \frac{4\,\sigma_f}{2\sqrt{2\ln 2}} \qquad (10)$$

This somewhat arbitrary decision is motivated by the fact that the full width at half maximum (FWHM) of a Gaussian can be expressed as:

$$FWHM = 2\sqrt{2\ln 2}\,\sigma \tag{11}$$

and since the maximum value of the Gaussian in this case is one, the expected probability of belonging to the detail component exceeds or equals the probability of belonging to the background at all spatial frequencies within the FWHM limits. To select a suitable range of spatial frequencies within which this could be expected, the FWHM is set to:

$$FWHM = 4\,\sigma_f \tag{12}$$

which leads to Eq. 10. This range of spatial frequencies, constrained within two standard deviations from the mean of the fitted Gaussian model, is selected simply based on the practical consideration that it includes most (approximately 95%) of the values of the Gaussian peak model while excluding the extreme parts of the tails. The standard deviation of the Gaussian selector function along the orientation axis is initially set to:

$$\sigma_{K,\vartheta} = \sigma_\theta\, b \tag{13}$$

where prior size *b* is an adjustable parameter with a default value of 0.5. The reason for incorporating the parameter *b* is to allow iterative inflation of the detail component spectral region starting from an initial smaller region, in a manner resembling segmentation algorithms based on region-growing strategies. This avoids the inclusion of noncontiguous spectral regions which are likely to originate from multiple different sources in the image. Initially, the Gaussian selector function is centered at the location$(\bar{f},\bar{\theta})$, as these values represent the expected mean orientation and mean spatial frequency of the detail component. Moreover, the Gaussian function is truncated to assign zero prior probability to spectral regions outside the specified spatial frequency cutoffs. The truncated Gaussian selector function with the initial parameter values can thus be expressed as:

$$K_0(f,\theta) = \begin{cases} e^{-\left(\frac{(f-\bar{f})^2}{2\sigma_{K,f}^2}+\frac{(\theta-\bar{\theta})^2}{2\sigma_{K,\theta}^2}\right)}, & f_L \le f \le f_H \\ 0, & \text{otherwise} \end{cases} \tag{14}$$

After the initial selector function has been formed, the initial spectral region of interest (ROI) $P_{D,0}$ representing the detail component is obtained by first computing the 'posterior' probabilities of each spectral element as:

$$X_0(f,\theta) = Z(f,\theta)\, K_0(f,\theta) \quad (15)$$

and then accepting all elements of the power spectrum for which $X_0 > 0.5$:

$$P_{D,0}(f,\theta) = \begin{cases} \overline{P}(f,\theta), & X_0(f,\theta) > 0.5 \\ 0, & \text{otherwise} \end{cases} \quad (16)$$

After $P_{D,0}$ has been obtained, the process proceeds in iterative manner. By default, the maximum number of iterations N_{max} is 10 and the convergence threshold τ is one degree, but these values can also be adjusted by the user. The iterations $i = 1 \ldots N_{max}$ proceed in the following steps:

1. Calculate mean orientation $\bar{\theta}_i$ and angular standard deviation $\sigma_{\theta,i}$ of the current spectral ROI $P_{D,i}$. Use $P_{D,0}$ during the first iteration.
2. Calculate the change in mean orientation $\Delta\bar{\theta}$ and change in angular standard deviation $\Delta\sigma_\theta$ relative to the previous values. If $\Delta\bar{\theta} < \tau$ and $\Delta\sigma_\theta < \tau$ or $i \geq N_{max}$, stop and return $P_{D,i}$.
3. Get updated selector function K_i according to Eq. 14 by substituting $\bar{\theta}$ with $\bar{\theta}_i$ and $\sigma_{K,\vartheta}$ with $\sigma_{\theta,i}$.
4. Get updated 'posterior' probabilities X_i according to Eq. 15 by substituting K_0 with K_i.
5. Get updated spectral ROI $P_{D,i+1}$. according to Eq. 16 by substituting X_0 with X_i.
6. Go to step 1.

Orientation analysis

For each spectral component, the distribution of orientations, normalized to assume the form of a probability density, is obtained as:

$$\Psi(\theta) = \frac{\sum_{f=0}^{f_{Nyquist}} P_c(f,\theta)}{\sum_{\theta=0}^{180^\circ} \sum_{f=0}^{f_{Nyquist}} P_c(f,\theta)} \quad (17)$$

where $P_C(f,\theta)$ is substituted either with the mixed component $P_M(f,\theta)$ or the detail component $P_D(f,\theta)$ spectral ROI. Angle doubling is performed to account for the axial nature of the data [22] and the mean orientation angle is afterwards divided by two. The following summary statistics are calculated using the corresponding functions of the CircStat toolbox [22]:

Mean orientation: The dominant orientation in degrees. Note that orientations repeat every 180 degrees i.e. the mean orientation always lies in the interval [0, 180) degrees.

Circular variance: A measure of spread of the orientation distribution. Circular variance is bounded in the interval [0, 1]. In the case of perfect anisotropy i.e. the whole distribution aligned along a single orientation, circular variance has a value close to zero. In the case of perfect isotropy i.e. the whole distribution spread out evenly around the half circle, circular variance has a value close to one. Note that high circular variance does not imply a uniform distribution but only the lack of any dominant direction.

Angular standard deviation: An alternative measure for the spread of the orientation distribution. Angular standard deviation is bounded in the interval $[0,\sqrt{2}]$. Similarly to circular variance, low values indicate anisotropy while high values indicate isotropy of the distribution.

Circular skewness: A measure of the symmetry of the distribution. Values close to 0 are indicative of a distribution that is symmetric around the mean orientation.

Circular kurtosis: A measure of peakedness. Large positive values indicate a strongly peaked distribution i.e. a distribution whose values are mostly concentrated to a limited number of orientation angles. Low values indicate a distribution whose values are 'smoothly' distributed across many different orientation angles.

Wavelength analysis

For each spectral component, the distribution of wavelengths is obtained by first converting the modified polar power spectrum into wavelength form. As the power spectrum is a density distribution function, expressing it in terms of wavelength λ rather than spatial frequency *f* is not simply a matter of making the substitution $f = 1/\lambda$ [48]. In addition to this substitution, the values in each spatial frequency bin need to be multiplied with a correction factor of $1/\lambda^2$ (or equivalently f^2) during the transformation. This correction is simply based on the requirement that total energy be conserved. The power spectrum as a function of wavelength can thus be expressed as:

$$\hat{P}(\lambda,\theta) = \frac{1}{\lambda^2} P_c\left(\frac{1}{\lambda},\theta\right) \tag{18}$$

where $P_C(f,\theta)$ is substituted either with the mixed component $P_M(f,\theta)$ or the detail component $P_D(f,\theta)$ spectral ROI. The wavelength distribution, normalized to assume the form of a probability density, is then obtained as:

$$\Lambda(\lambda) = \frac{\sum_{\theta=0}^{180^\circ} \hat{P}(\lambda,\theta)}{\sum_{\lambda_{min}}^{\lambda_{max}} \sum_{\theta=0}^{180^\circ} \hat{P}(\lambda,\theta)} \tag{19}$$

Linear interpolation is then performed to obtain equidistant spacing of data points. The following summary statistics are then calculated:

Mean wavelength: The mean of the wavelength distribution.

Median wavelength: The median of the wavelength distribution.

Mode: The mode of the wavelength distribution.

Standard deviation: The standard deviation of the wavelength distribution.

Supplementary simulation methods

Generation of synthetic phase contrast microscopy images of cell clusters

Synthetic images with bright targets on a dark background, resembling clusters of cells, were generated using a custom MATLAB script. An image size of 1024 x 1024 pixels, 10x magnification and an image pixel size of 0.68 μm x 0.68 μm were used as the basis of the images. The targets were randomly located two-dimensional Gaussians with varying sizes and orientations. First, several parameter values were obtained for each image by random sampling from uniform distributions. The maximum number of targets per image was sampled from the interval [10, 100]. The mean width of the targets i.e. the full width at half maximum (FWHM) of the Gaussians, was sampled from the interval (10, 50) μm. The standard deviation of the target width was sampled from the interval (0, 20) μm. The aspect ratio of the targets, i.e. the ratio of the target length to the target width, was sampled from the interval (1, 5). Parameters for the von Mises orientation distribution i.e. mean direction and concentration parameter kappa were sampled from the intervals (0, π) and (0, 100), respectively.

For each image, targets were located one by one, first sampling the center of each target from a uniform distribution and the direction of the target from a von Mises distribution with the given mean direction and concentration parameters using the corresponding function from the CircStat toolbox [22]. Directions were converted to orientations by subtracting π from all direction values larger than or equal to π. The width of each target was then sampled from a normal distribution having the previously obtained mean and standard deviation parameters. Widths less than 5 μm were rejected and the sampling was repeated to ensure that each target had an acceptable number of pixels. Target length was then calculated on the basis of the target width and aspect ratio values. Points belonging to the target were defined as the set of pixels located within an elliptical region at most two standard deviations from the center of the Gaussian. If any of these pixels were located in a forbidden region, the target was rejected and the random placement was repeated. Otherwise, the target was accepted. Initially, the forbidden region comprised all points located within ten pixels from the edges of the image. Whenever a target was accepted, all of the pixels belonging to the target were added to the forbidden region, preventing placement of new targets on existing ones. The target placement process was continued until the maximum number of targets had been placed or more than 1000 consecutive iterations were rejected. The synthetic images were output in TIF format and the orientation and size distributions of the targets were saved for performance calculations. In total, 1000 images were generated.

Generation of synthetic fluorescence microscopy images of cells with intracellular structures

Synthetic images of cells with intracellular fibrils were generated using the MATLAB implementation (version 1.0) of SimuCell [24] and custom MATLAB scripts. An image size of 1024 x 1024 pixels, 40x magnification and an image pixel size of 170 nm x 170 nm were used as the basis of the images. For each image, SimuCell was first used to create a single cell with the cytoplasm stained red and the nucleus blue. Random nuclear placement was constrained within two pixels from the midpoint of the image. If any parts of the cell were located less than ten pixels away from the image edge, the cell generation procedure was repeated. For the nuclei, the default nuclear model was used with the following parameters: radius 10 μm, eccentricity 0.5, randomness 0.1, mean intensity 0.5 and intensity standard deviation 0. For the cytoplasms, a nuclear-centric model was used with the following parameters: radius 50 μm, eccentricity 0.7, randomness 0.7, mean intensity 0.3 and intensity standard deviation 0. Default compositing with a container weight of zero was used for all images.

Intracellular fibrillar structures were generated for each cell using a MATLAB script. These structures resembled the myofibrils of hiPSC-CMs in our test sets 1 and 2, stained with anti-cardiac Troponin T (red), anti-Myosin binding protein C3 (green) and DAPI (blue). In short, each fibril was constructed from a varying number of smaller subunits, each a two-dimensional Gaussian, organized at different orientations and varying intervals along a linear segment within the cell. First, several parameter values were obtained for each cell by random sampling from uniform distributions. The aspect ratio of the subunits, i.e. the ratio of the subunit length to the subunit width, was sampled from the interval (1, 5). A fixed subunit width of 1.3 μm, corresponding to the full width at half maximum (FWHM) of the Gaussians along the longitudinal direction of the fibril, was used for all cells. Subunit length, i.e. the FWHM of the Gaussians along the transverse direction of the fibril, thus ranged from 1.3 μm to 6.5 μm. Mean and standard deviation of the normally distributed distances between neighboring subunits along a fibril were sampled from the intervals (1.5, 2.5) μm and (0, 0.2) μm, respectively. Minimum spacing between fibrils was sampled from the interval (subunit length, subunit length + 10 μm). Parameters for the von Mises orientation distribution i.e. mean direction and concentration parameter kappa were sampled from the intervals (0, π) and (0, 100), respectively.

Fibrils were located one by one, first sampling the midpoint of the fibril from a uniform distribution and the direction of the fibril from a von Mises distribution with the given mean direction and concentration parameters using the corresponding function from the CircStat toolbox [22]. Directions were converted to orientations by subtracting π from all direction values larger than or equal to π. Fibril length was then sampled from a normal distribution with an initial mean of 50 μm and an initial standard deviation of 10 μm. End points of the fibril were then calculated based on the known midpoint, length and orientation. Points along the entire length of the fibril were

subsequently computed using linear interpolation with tenfold upsampling. The resulting region was then thickened by dilating using a square structuring element of ones with size 3x3 pixels. If any of the points of this region were located in a region of the image that is forbidden for fibrils, the fibril was rejected. Otherwise, the fibril was accepted. Initially, the forbidden region consisted of all pixels outside the cell or within the nucleus, dilated by a square structuring element of ones with size equal to twice the subunit length. The dilation was performed to avoid parts of the fibrils extending out from the cell or into the nucleus. When a fibril was accepted, the region consisting of the interpolated points of the fibril was first dilated using a square structuring element of ones with size equal to the given fibril spacing. This region was then added to the existing forbidden region, preventing the placement of new fibrils less than the given fibril spacing away from existing ones. If more than 10 000 consecutive iterations were rejected, the mean and the standard deviation of the fibril length were decreased by 10 % to fit more fibrils into the cell. The fibril placement process was continued until either a maximum number of 20 fibrils were accepted, the mean fibril length became shorter than the mean subunit-to-subunit distance times 15 or more than 100 000 consecutive iterations were rejected.

After the locations of the fibrils had been chosen, the image was formed by locating the fibril subunits. The subunits were located fibril by fibril, starting from one end of each fibril and proceeding towards the other end in steps whose sizes were sampled from a normal distribution with the given mean and standard deviation for subunit-to-subunit distance. The orientation of each Gaussian was sampled from a von Mises distribution having a mean orientation equal to the orientation of the fibril in question, and a concentration parameter equal to the concentration parameter of the fibrils, again using the corresponding function from the CircStat toolbox [22]. As in the case of the complete fibrils, π was subtracted from directions larger than or equal to π. The resulting image, consisting of Gaussians organized into fibrils, was used to construct the green channel of the synthetic image.

To add some interference to the green channel, resulting from the nucleus and the cytoplasm in the blue and red channels, respectively, an RGB image was first formed directly from the three color channels. The final green channel image was formed by converting the RGB image to grayscale format using MATLAB’s *rgb2gray* function i.e. by eliminating the hue and saturation information while retaining only the luminance. As the relative intensities of the red, green and blue channels in the RGB image were 0.3, 1 and 0.5, respectively, the high intensity features of the resulting image represent the target fibrils, while weaker background signals originate from the cytoplasm and the nucleus. The motivation for this mixing operation is that in real images, the objects of interest are often accompanied by other interfering features, which arise e.g. in the case of fluorescence microscopy from unspecific binding of antibodies to different parts of the cell. The

final RGB image was then constructed from the green channel image with the added interference, and the original red and blue channel images created by SimuCell. Finally, the red, green and blue channels of the image were rescaled to have relative intensities of 0.8, 1 and 0.9, respectively, in accordance with observations made from our test set images. The synthetic images were output in TIF format. In total, 1000 images were generated.

Degradation of synthetic images

Synthetic images were blurred by filtering with with MATLAB's *imfilter* function and a Gaussian filter having a prescribed standard deviation. The standard deviation value was adjusted to obtain images with varying degrees of blurring. The dimensions of the square filter were set equal to six times the standard deviation. Image boundaries were handled by assuming values outside the image to equal the value of the nearest pixel on the image border. Blurring was applied separately to each color channel in the case of RGB images.

Additive zero mean white Gaussian noise and Poisson noise were added using MATLAB's *imnoise* function. The variance of the white Gaussian noise was adjusted to obtain images with different amounts of white Gaussian noise. The amount of Poisson noise was adjusted by first scaling the image intensity values down by division with the desired noise level and then scaling the values up with the same noise level value after the noise generation step. Noise was separately generated for each color channel in the case of RGB images.

Supplementary experimental methods

Patient-specific human iPSC lines and culturing

The collection of biopsies for generating patient specific human induced pluripotent stem cell (hiPSC) lines was approved by the ethical committee of Pirkanmaa Hospital District (Aalto-Setälä R08070) and written informed consent was obtained from all the donors. Human iPSC-lines have been established by sendai viral (CytoTune® iPS reprogramming kit, Thermo Fisher Scientific, Waltham, MA, USA) or retroviral transfection of *OCT3/4*, *SOX2*, *KLF4* and *c-MYC* [26]. Characterization of hiPSCs included confirmation of mutations by qPCR, karyotype analysis, confirmation of gene expression by PCR and protein expression by immunocytochemistry. The pluripotency of hiPSC lines was confirmed by EB-formation and teratoma formation in mice.

Human iPSCs were cultured on mitomycin-C inactivated mouse embryonic fibroblasts (26,000 cells/cm2, CellSystems Biotechnologie Vertrieb GmbH, Troisdorf, Germany), which act as feeder cells to support the growth of undifferentiated hiPSCs and to maintain their pluripotent state in hiPSC maintenance medium (KO-DMEM (Thermo Fisher Scientific) base, 20% KO-SR (Thermo Fisher Scientific), 1% non-essential amino acids (NEAA, Lonza Group Ltd, Basel, Switzerland), 2mM GlutaMax (Thermo Fisher Scientific), 50 U/ml penicillin/streptomycin (Lonza), 0.1 mM 2-mercaptoethanol (Thermo Fisher Scientific) and 4 ng/ml basic fibroblast growth factor (bFGF, Peprotech, Rocky Hill, NJ, US)). Medium was changed three times per week. The undifferentiated state of hiPSCs was confirmed by morphologic analysis with Nikon Eclipse TS100 phase contrast microscope (Nikon Instruments Europe B.V. Amstelveen, The Netherlands) with Altra-Cell-D-Bundle camera (Olympus Corporation, Tokyo, Japan) and undifferentiated colonies were passaged weekly on new MEFs. MEF feeder cell layers were manually removed by scraping with a pipette tip and treated with 1 mg/ml Collagenase IV (Thermo Fisher Scientific). Remaining attached hiPSC colonies were transferred to a new culture plate.

Cardiomyocyte differentiation

Differentiation into cardiomyocytes was carried out by co-culturing hiPSCs (50000 cells/cm2) with murine visceral endoderm-like (END-2) cells (prof. Mummery, Humbrecht Institute, Utrecht, The Netherlands) [25]. END-2 –cells were mitomycin-C (Sigma Aldrich, St. Louis, MO, USA) inactivated (5 µl/ml, 3h), trypsinized (Lonza) and plated on 12-well-plate (Nunc) 175,000 cells/well in DMEM-F12 (Thermo Fisher Scientific) based medium (7.5% fetal bovine serum (FBS, Biosera, Boussens, France), 1% NEAA (Lonza), 2 mM GlutaMax (Thermo Fisher Scientific) and 50 U/ml penicillin/streptomycin (Lonza)). END-2 –cells were let to attach in 37°C overnight. 0% KO-SR KO-DMEM (Thermo Fisher Scientific) based medium (1% NEAA (Lonza), 2mM GlutaMax (Thermo Fisher Scientific), 50 U/ml penicillin/streptomycin (Lonza) and 0.1mM β-mercaptoethanol (Thermo

Fisher Scientific)) was changed to END-2 cells an hour before plating the hiPSCs. MEFs were removed as described above. hiPSC -colonies were transferred on END-2 –cells ~30 colonies/well with minimal amount of culture medium. Cells were co-cultured in 37°C and 5% CO_2. The 0% KO-SR -medium was changed at days 5, 8 and 12. After 15 days of culturing, the medium was changed to 10% KO-SR KO-DMEM (Thermo Fisher Scientific) based medium (10% KO-SR (Thermo Fisher Scientific), 1% NEAA (Lonza), 2mM GlutaMax (Thermo Fisher Scientific), 50 U/ml penicillin/streptomycin (Lonza) and 0.1mM β-mercaptoethanol (Thermo Fisher Scientific)) and changed 3 times a week. Cells were monitored with phase-contrast microscopy (Nikon Eclipse TS100 microscope, Imperx IGV-B1620M-KC000 camera, JAI Camera Control Tool software). hiPSCs formed spontaneously beating clusters after 15 days of co-culturing.

Dissociation of beating areas

Beating clusters were cut and isolated with scalpel and dissociated [27]. Beating areas were washed in Low-Ca buffer (12ml 1M NaCl, 0.54ml 1M KCl, 0.5ml 1M $MgSO_4$, 0,5ml 1M Na pyruvate, 2ml 1M glucose, 20ml 0,1M taurine and 1ml 1M HEPES (pH adjusted to 6.9 with NaOH)) at room temperature (RT) for 30 minutes and treated with buffer (12ml 1M NaCl, 3µl 1M $CaCl_2$, 0.54ml 1M KCl, 0.5ml 1M $MgSO_4$, 0.5ml 1M Na pyruvate, 2ml 1M glucose, 20ml 0.1M taurine, 1 mg/ml collagenase A (Roche Diagnostics, Basel, Switzerland) and 1ml 1M HEPES (pH adjusted to 6.9 with NaOH)) including collagenase A (Roche Diagnostics) at 37 ˚C for 45 minutes. Cells were incubated in KB medium (3ml 1M K_2HPO_4, 8.5ml 1M KCl, 2mmol/l Na_2ATP, 0.5ml 1M $MgSO_4$, 0.1ml 1M EGTA, 0.5ml 1M Na pyruvate, 2ml 1M glucose, 5ml 0.1M creatine and 20ml 0.1M taurine, pH 7.2) (20µl of 1M glucose was added per 1ml of KB-medium before use) at RT for one hour. Washed beating areas were resuspended in EB-medium (20% FBS (Biosera) in KO-DMEM (Thermo Fisher Scientific), 1% NEAA (Lonza), 2mM GlutaMax (Thermo Fisher Scientific), 50 U/ml penicillin/streptomycin (Lonza)) by pipetting up and down several times against the bottom of the dish.

Flexcell® experimenting

Dissociated cardiomyocytes were plated on Uniflex®-plates (Dunn Labortechnik, Asbach, Germany) and 0.1% gelatin (type A from porcine, Sigma Aldrich) coated 13mm coverslips (No.1, VWR, PA, USA) and let to attach at 37˚C for 45 minutes before loading up to the final amount of EB-medium (3 ml/well on Uniflex-plates and 800µl/well on 4-well-plates). Cells were cultured three days in EB-medium before starting the stretching on Flexcell® (Burlington, NC, USA) apparatus with arctangle loading stations. Cells were stretched with sinusoidal cyclic stress of 7-10% elongation for 4-14 days. EB-medium was changed three times a week. After finishing the stretching, samples were fixed immediately with 4% PFA (Sigma Aldrich) by the double fluorescence protocol (see below).

Peripheral sensory neuron differentiation

PSN images 10-15: Differentiation protocol was obtained from [30]. Mouse bone marrow-derived stromal cells (PA6, RIKEN Cell Bank, Tsukuba, Japan) were used as feeders. PA6 cells were thawn, cultured on gelatin coated 75 cm2 cell culture flasks in KO-DMEM based PA6 medium (10% FBS (Biosera), 0.1mM NEAA (Lonza), 2mM Glutamax (Thermo Fisher Scientific), 50 U/ml penicillin/streptomycin (Lonza)) and inactivated by irradiation. PA6 -cell layer was plated on cell culture wells at least one day before adding undifferentiated hiPSCs.

Human iPSC colonies were transferred from MEFs to PA6 feeder cell line as described earlier. Approximately 20 µl of cell suspension (approximately 60 000 cells) was used per cell culture well and transferred to differentiation plate. Induction phase was done co-culturing iPS colonies with 95% confluent PA6 feeder cell layer in 0.1% gelatin coated 6-well cell culture plates. GMEM (BHK-21) based induction medium (10% KO-SR (Thermo Fisher Scientific), 0.1mM NEAA (Lonza), 2mM Glutamax (Thermo Fisher Scientific), 1mM Na-pyruvate (Sigma Aldrich), 50 U/ml penicillin/streptomycin (Lonza), 0.1mM β-mercaptoethanol (Thermo Fisher Scientific)) was changed three times a week (except the first change which was done four days after plating to ensure cell attachment).

After 12 days of culture in induction medium, cell colonies with distinct round and solid shape were visible. These colonies were cut off with microsurgical scalpel under microscope and moved to low-cell bind 12-well culture plate (Nunc) in DMEM/F12 (Thermo Fisher Scientific) based Neurosphere medium (1% B27-supplement (Thermo Fisher Scientific) 2mM Glutamax (Thermo Fisher Scientific) 1mM Penicillin/Streptomycin (Lonza) 20 ng/ml bFGF (Peprotech)). 20 spheres were cultured per 1ml of neurosphere medium which was changed twice a week. Neurospheres were cut with microsurgical scalpel and micro lancet needle into smaller pieces every week. The cells were cultured in this phase for four to eight weeks.

Neurospheres were dissociated with trypsin (Lonza) for 5-10min in +37°C. Trypsin was neutralized by rinsing with DMEM-F12 (Thermo Fisher Scientific) based DRG medium (1 % B27-supplement (Thermo Fisher Scientific) 2mM Glutamax (Thermo Fisher Scientific) 1mM Penicillin/Streptomycin (Lonza) 10 ng/ml neural growth factor (NGF, R&D Tocris)). Finally, spheres were plated onto PA6 feeders on either gelatin (porcine type-A, Sigma Aldrich) or laminin-1 (murine Engelberth-Holm-Swarm, Sigma Aldrich) coated culture plates when they had clearly started to dissociate. Medium was changed twice a week.

PSN images 1-9: Differentiation and neural induction protocol was obtained from [31]. MEFs were removed and the same amount of hiPSC suspension, as above, was transferred to new differentiation plate. Cells were plated on Matrigel-treated (BD biosciences, Franklin Lakes, NJ, USA) dishes (17.5 mg/cm2) at a density of ~60,000 cells/cm2 in the presence of MEF-conditioned hiPSC maintenance medium (see above) supplemented with 10 ng/ml bFGF (Peprotech) and 10 µM ROCK inhibitor (R&D Systems). Neural differentiation was initiated when the cells were almost confluent using KO-DMEM (Thermo Fisher Scientific) based KSR medium (15% KO-SR (Thermo Fisher Scientific), 1mM l-glutamine, 0.1mM NEAA (Lonza) and 0.1mM β-mercaptoethanol (Thermo Fisher Scientific)). To inhibit SMAD signaling, 100nM LDN-193189 (Stemgent, Cambridge, MA, USA) and 10µM SB431542 (Calbiochem, Merckbiosciences) were added on days 0–5. Medium was changed daily, and DMEM-F12 (Thermo Fisher Scientific) based N2 supplement (Thermo Fisher Scientific) -medium (containing 1mM Penicillin/Streptomycin (Lonza)) was added in increasing 25% increments every other day starting on day 4 (100% N2 on day 10). Sensory neuron induction was initiated with the addition of the three inhibitors, 3µM CHIR99021 (Merck Millipore), 10µM SU5402 (Merck Millipore) and 10µM DAPT (Stemgent), on days 2–10. Long-term culture medium (N2 supplement -medium) containing 25 ng/ml human-β-nerve growth factor (h-βNGF, R&D systems), -brain-derived neurotrophic factor (h-BDNF, R&D systems) and -glial cell-derived neurotrophic factor (h-GDNF, R&D systems).

Immunocytochemistry and image acquisition

Cells were stained by double fluorescence protocol: Culture-medium was aspirated and the cells were washed with 1xPBS (0.01M, pH7.4, Dulbecco's phosphate buffer saline, Lonza) two times at RT for 5min. Cells were fixed with 4% paraformaldehyde (PFA, Sigma-Aldrich) for 20min at RT. Cells were washed again two times with 1xPBS for 5min. Uniflex® membranes were cut off from plate after fixing and washes. Blocking and permeabilizing was done by applying PBS based solution (10% NDS (normal donkey serum, Merck Millipore), 0.1% TritonX-100 (Sigma Aldrich), 1% BSA (Sigma Aldrich)) for 45min at RT. Cells were washed with PBS based solution (1% NDS, 0.1% TritonX-100, 1% BSA) before adding primary antibodies. Primary antibodies of cardiac troponin T (goat IgG, 1:2000, Abcam), alpha actinin (mouse IgG, 1:1500, Sigma Aldrich) and myosin binding protein C (mouse IgG, 1:400, Santa Cruz) were used. Mixtures of primary antibodies were prepared in PBS based solution (1% NDS, 0.1% TritonX-100, 1% BSA). Cells were incubated with primary antibody mixtures in +4°C overnight. Next day, cells were washed three times with 1% BSA in PBS for 5min before applying secondary antibodies. Alexa Fluor 488 (anti mouse, 1:800, Thermo Fisher Scientific) and Alexa Fluor 568 (anti goat, 1:800, Thermo Fisher Scientific) were used. Secondary antibody mixtures were prepared in 1% BSA in PBS. Cells were incubated with secondary antibody mixtures for 1hr in RT. Cells were washed three times with 1xPBS for 5min and two times with phosphate buffer working solution (0.01M

Na_2HPO_4/NaH_2PO_4, pH7.4) for 5min. Vectashield (Vector Laboratories Inc, Burlingame, CA, USA), containing 4',6-diamidino-2-phenylindole (DAPI) was used to stain nuclei and mount the samples on standard microscope glass. The stressed samples on Uniflex®-membranes were cut off the 6-well-plates and placed always on the same direction on the microscope glass for maintaining the direction relative to the stress applied for microscoping.

Imaging of immunostained samples was carried out with Zeiss (Carl Zeiss AG, Oberkochen, Germany) AxioScope A1 upright fluorescent microscope and Zeiss AxioCam MRc5 camera. Using 2x2 binning, the effective pixel size of the image sensor was 6.8µm x 6.8µm. The microscope was operated with Zeiss' Zen 2012 software via PC. 20x and 40x air objectives were used for imaging. Live PSN cells were imaged at the age of around 20d (depending on differentiation method) with Nikon (Tokyo, Japan) Eclipse TS100 inverted phase contrast microscope with Imperx IGV-B1620M camera. 10x air objective was used without binning (1x1) pixel size of the image sensor being 7.4µm x 7.4µm.

Symbols

N_{max}: maximum number of iterations parameter
W: resolution parameter
L: segment length parameter
α: significance level parameter
ε: angular tolerance parameter
τ: convergence threshold parameter
b: prior size parameter
N_S: number of segments averaged during WOSA
$F(u, v)$: power spectrum expressed as a function of spatial frequencies u and v in Cartesian coordinates
$P(f, \theta)$: power spectrum expressed as a function of spatial frequency f and orientation θ in polar coordinates i.e. polar power spectrum
$f_{Nyquist}$: Nyquist frequency
N_{uv}: number of spatial frequency bins in the Cartesian power spectrum
N_θ: number of angle bins in the polar power spectrum
N_f: number of spatial frequency bins in the polar power spectrum
$\overline{P}(f, \theta)$: adjusted polar power spectrum
$R(f, \theta)$: normalized polar power spectrum
$S(f, \theta)$: background polar power spectrum
$Z(f, \theta)$: probabilistic polar power spectrum

f_L: lower spatial frequency cutoff

N_L: number of low spatial frequency spectral bins to exclude starting from zero frequency

f_H: higher spatial frequency cutoff

$P_M(f, \theta)$: mixed component

$\bar{\theta}$: initial mean orientation of the detail component

$\bar{f}$: mean of the Gaussian detail component model

σ_f: standard deviation of the Gaussian detail component model

σ_θ: initial angular standard deviation of the detail component

$K(f, \theta)$: Gaussian selector function

$X(f, \theta)$: probabilistic detail component

$\sigma_{K,f}$: standard deviation of the Gaussian selector function along the spatial frequency axis

$\sigma_{K,\theta}$: standard deviation of the Gaussian selector function along the orientation axis

$P_D(f, \theta)$: detail component

$\Psi(\theta)$: orientation distribution

$\hat{P}(\lambda, \theta)$: polar power spectrum as a function of wavelength λ and orientation θ in polar coordinates

$\Lambda(\lambda)$: wavelength distribution

Supplementary references

[41] Field DJ. Relations between the statistics of natural images and the response properties of cortical cells. J Opt Soc Am A. 1987;4:2379-94.

[42] Tolhurst DJ, Tadmor Y, Chao T. Amplitude spectra of natural images. Ophthalmic Physiol Opt. 1992;12:229-32.

[43] Ruderman DL. Origins of scaling in natural images. Vision Res. 1997;37:3385-98.

[44] Field DJ, Brady N. Visual sensitivity, blur and the sources of variability in the amplitude spectra of natural scenes. Vision Res. 1997;37:3367-83.

[45] Hsiao WH, Millane RP. Effects of occlusion, edges and scaling on the power spectra of natural images. J Opt Soc Am A Opt Image Sci Vis. 2005;22:1789-97.

[46] Ferraioli L, Hewitson M, Congedo G, Nofrarias M, Hueller M, Armano M, et al. Quantitative analysis of LISA pathfinder test mass noise. Phys Rev D Part Fields. 2011;84:122003.

[47] Johnson PE, Long DG. The probability density of spectral estimates based on modified periodogram averages. IEEE Trans Signal Process. 1999;47:1255-61.

[48] Soffer BH, Lynch DK. Some paradoxes, errors, and resolutions concerning the spectral optimization of human vision. Am J Phys. 1999;67:946-53.
